# Effectiveness of psychological interventions for adult survivors of the 2023 Kahramanmaraş earthquakes: a systematic review and meta-analysis

**DOI:** 10.3389/fpsyg.2025.1696103

**Published:** 2025-12-17

**Authors:** Metin Çınaroğlu

**Affiliations:** Department of Psychology, Faculty of Administrative and Social Science, İstanbul Nişantaşı University, Istanbul, Türkiye

**Keywords:** Kahramanmaraş, psychological interventions, PTSD, depression, anxiety, cognitive behavioral therapy, meta-analysis

## Abstract

**Background:**

The February 6, 2023 Kahramanmaraş earthquakes were among the most devastating disasters in Türkiye’s history, leaving millions affected and exposing survivors to elevated risks of post-traumatic stress disorder (PTSD), depression, and anxiety. While numerous psychological interventions have been deployed in the aftermath, their effectiveness for this population has not been systematically evaluated.

**Objective:**

This study synthesized evidence on the effectiveness of psychological interventions delivered to adult survivors of the Kahramanmaraş earthquakes, with a focus on PTSD, depression, and anxiety outcomes.

**Methods:**

A systematic review and meta-analysis was conducted in accordance with PRISMA guidelines and registered on PROSPERO (CRD420251102991). Eligible studies were randomized or quasi-experimental trials assessing structured psychological interventions (e.g., trauma-focused cognitive behavioral therapy [TF-CBT], psychoeducation, spiritually integrated approaches, telepsychiatry, and virtual reality–based programs) among earthquake-exposed adults. Primary outcomes were PTSD, depression, and anxiety, measured with validated instruments. Random-effects models were used to pool effect sizes.

**Results:**

Nine peer-reviewed studies (*N* = 435 participants) were identified. Interventions included TF-CBT, Islamic TF-CBT, spiritually oriented logotherapy, VR-based trauma therapy, CBT-based psychoeducation, and telepsychiatry. Meta-analyses indicated large pooled effects favoring interventions: PTSD (Hedges’ *g* = −2.6, 95% CI [−4.0, −1.3]), depression (*g* = −1.27, 95% CI [−1.73, −0.81]), and anxiety (*g* = −1.18, 95% CI [−1.55, −0.82]). Benefits were consistent across individual, group, and online formats. Culturally adapted approaches appeared to produce meaningful improvements, although the evidence is limited. Follow-up assessments across studies ranged widely (1–12 months). Short-term follow-up data (1–3 months) generally indicated maintained symptom reductions, whereas evidence from longer-term follow-up (6–12 months) was limited to a small number of studies and should therefore be interpreted cautiously.

**Conclusion:**

Structured psychological interventions–especially trauma-focused CBT, culturally adapted therapies, and psychoeducation–significantly alleviated PTSD, depression, and anxiety among adult survivors of the Kahramanmaraş earthquakes. Group-based and telehealth modalities proved feasible and effective, expanding access when in-person services were disrupted. Despite promising results, evidence remains limited by small sample sizes, heterogeneity, and short follow-up. Future research should address long-term outcomes, functional recovery, and broader populations, including children and first responders.

**Systematic review registration:**

https://www.crd.york.ac.uk/PROSPERO/view/CRD420251102991, identifier CRD42025837569

## Introduction

Earthquakes are among the most devastating natural disasters, often exacting not only a toll of lives and physical destruction but also leaving deep psychological scars on survivors (Spiridonov et al., 2025). Globally, exposure to major earthquakes has been consistently linked with elevated rates of mental health disorders (Bertinelli et al., 2023). PTSD is frequently cited as the most common psychiatric consequence of such disasters (Çitak and Dadandı, 2024; Fong et al., 2022; Karamustafalıoğlu et al., 2023). Meta-analyses indicate that nearly one in four earthquake survivors develop PTSD, though reported prevalence varies widely with context and study methods (Hosseinnejad et al., 2022; Paudel et al., 2025; Tahernejad et al., 2023). For instance, population-based studies after the 1999 Marmara earthquake in Türkiye found PTSD prevalence ranging from about 8% to 63% of survivors, and major depression from 11% to 42% (Aker, 2006). Similarly, a systematic review of the 2010 Haiti earthquake reported that approximately 28% of survivors experienced severe PTSD symptoms, alongside 32% with severe depression and 20% with severe anxiety (Dube et al., 2018). Even years after a major quake, a substantial minority of survivors may continue to suffer chronic psychological distress if left unaddressed (Çınaroğlu et al., 2025). These patterns underscore that the mental health impact of earthquakes is both profound and enduring worldwide.

### Psychological sequelae of earthquakes: PTSD, depression, and anxiety

Post-traumatic stress disorder, depression, and anxiety are the most frequently reported psychological consequences of major earthquakes. PTSD is the prototypical trauma-related disorder, characterized by intrusive recollections, avoidance, and hyperarousal. Meta-analytic and longitudinal studies show prevalence estimates ranging from 4% to 60%, shaped by exposure severity, time since the disaster, and sociodemographic factors (Farooqui et al., 2017; Tang et al., 2017; İskifoğlu and Kara, 2024). Long-term follow-ups confirm that post-earthquake PTSD may persist for years; for example, Goenjian et al. (2011) found that nearly 9% of adolescents continued to experience moderate-to-severe PTSD 32 months after the Parnitha earthquake, while Zhang et al. (2012) identified depressive symptoms and female gender as significant predictors of PTSD severity 18 months after the Wenchuan earthquake.

Depression frequently co-occurs with PTSD, contributing to chronic impairment in functioning and well-being. In a 10-years Turkish follow-up, Karamustafalıoğlu et al. (2023) reported enduring depression and trauma symptoms, while Lu et al. (2023) found that prenatal exposure to the Tangshan earthquake predicted higher depressive symptoms and suicidal ideation in adulthood. Early post-disaster studies have also documented high depressive rates across populations: Sharma et al. (2021) reported 43% prevalence of depression among Nepalese youth, and Sert et al. (2025) found severe depressive and anxious symptoms among Turkish nurses following the 2023 Kahramanmaraş earthquakes, even in those indirectly affected. Similarly, Xu et al. (2023) demonstrated strong comorbidity between depressive and PTSD symptom clusters in adolescent survivors of the Wenchuan and Ya’an earthquakes.

Anxiety is another dominant response, reflecting persistent fear, vigilance, and loss of perceived safety. In Türkiye et al. (2024) observed significantly elevated anxiety among young adults after the Kahramanmaraş earthquakes, while Uğur et al. (2021) found that anxiety sensitivity and perceived stress predicted peritraumatic dissociation in acute stress disorder patients, increasing later PTSD risk. International studies report comparable findings: Gerstner et al. (2020) identified heightened anxiety among Ecuadorian adolescents 9 months after the Muisne earthquake, and Tang et al. (2020) showed that separation anxiety and panic symptoms were prevalent among Chinese adolescents 3 years post-disaster, with peer bullying, poverty, and parental absence as key predictors. Collectively, PTSD, depression, and anxiety constitute interrelated yet distinct outcomes that jointly define the psychological aftermath of earthquakes, underscoring the need for interventions that address the full spectrum of post-disaster distress.

Earthquake-related psychological outcomes are also interrelated in ways that have been extensively documented in the literature. PTSD, depression, and anxiety frequently co-occur following disaster exposure, sharing common pathways such as intrusive rumination, perceived threat, loss of control, and heightened physiological arousal (Farooqui et al., 2017; Xu et al., 2023). Meta-analytic evidence demonstrates strong correlations between PTSD symptom clusters and depressive affect, with hyperarousal and negative alterations in cognition predicting subsequent increases in depressive symptoms (Goenjian et al., 2011; Hosseinnejad et al., 2022). Post-disaster anxiety is similarly linked to both PTSD and depression through mechanisms such as catastrophic thinking, vigilance, and stress sensitivity (Gerstner et al., 2020; Tang et al., 2020). Positive and negative affect–assessed in several of the included interventions–are well-established emotional indicators associated with trauma recovery trajectories, where lower positive affect and elevated negative affect predict more severe PTSD and depressive distress (Karamustafalıoğlu et al., 2023). Likewise, resilience functions as a moderating or buffering factor, attenuating the impact of trauma exposure on these symptom dimensions (Haktanir and Kurnaz, 2024). Together, this evidence justifies evaluating these variables within a unified intervention framework, as improvements in one domain often coincide with concurrent benefits across related psychological outcomes.

This study is conceptually grounded in the Conservation of Resources (COR) theory (Hobfoll, 1989, 2001), one of the most influential frameworks in trauma and disaster psychology. COR theory posits that psychological distress arises when individuals face actual resource loss, threats to valued resources, or insufficient resource gains following adversity. Earthquakes are characterized by acute and massive depletion of core resources–including safety, housing stability, social support, emotional equilibrium, and a sense of control. Hobfoll’s (2001) expanded model further emphasizes “resource caravans” (clusters of interrelated resources) and “resource passageways” (environmental conditions that enable or hinder resource recovery), which are highly relevant in post-disaster contexts marked by displacement, infrastructure loss, and community disruption. Psychological interventions aim to restore these depleted resources by strengthening coping capacity, rebuilding social and cognitive resources, enhancing emotion regulation, and reinstating a sense of safety and mastery. Thus, improvements in PTSD, depression, anxiety, affect, and resilience observed across intervention studies can be conceptualized as the rebuilding of disrupted resource systems, providing a coherent theoretical foundation for evaluating post-earthquake mental-health interventions.

Given this substantial burden of trauma-related disorders following earthquakes, the implementation of effective post-disaster psychological interventions is a global public health priority (Semerci and Uzun, 2023). Over the past two decades, an expanding evidence base has examined interventions to mitigate disaster-related mental health problems. Randomized trials and meta-analyses in diverse disaster settings have indicated that structured psychological treatments can significantly reduce PTSD, depression, and anxiety symptoms compared to no treatment or usual care (Dai et al., 2016; Jahangiri et al., 2020). TF-CBT in particular has the strongest evidence and is recommended as a first-line treatment for PTSD by international guidelines (Mavranezouli et al., 2020). For example, a recent meta-analysis of 12 randomized trials in natural disaster survivors (primarily earthquakes) found that trauma-focused interventions (mostly CBT-based) led to large short-term reductions in PTSD symptoms (pooled Hedges’ *g* = 1.44 versus passive controls) and maintained significant improvements at follow-up (*g* = 0.59 after ∼2 months, Kip et al., 2024). Other therapeutic modalities – such as EMDR, narrative exposure therapy, interpersonal psychotherapy, and group-based interventions – have also been applied after disasters with generally positive outcomes. Indeed, a recent comprehensive review of earthquake-related PTSD interventions concluded that psychological treatments were associated with substantial clinical improvements, including a large overall effect size for PTSD symptom reduction and a moderate effect for comorbid depression (Woods et al., 2025). Alongside formal therapies, broad psychosocial support strategies (for example, psychological first aid and community outreach programs) are commonly deployed in the immediate aftermath to promote safety, coping, and social support, though their specific impacts are less easily quantified (Lopes et al., 2014). Overall, the literature suggests that timely, evidence-based mental health interventions can play a critical role in promoting recovery in disaster-stricken populations.

Despite the growing global evidence, significant knowledge gaps remain regarding what works best for specific disasters and cultural contexts. The catastrophic earthquakes that struck southeastern Türkiye on 6 February 2023 – referred to here as the Kahramanmaraş earthquakes – presented an unprecedented mental health challenge for the region. Affecting over 13 million people across 11 provinces, these twin earthquakes (Mw 7.7 and 7.6) caused more than 50,000 deaths and mass displacement (Toprak S. et al., 2025). In the aftermath, extraordinarily high levels of psychological trauma have been observed among adult survivors. One large survey found that over half (55.2%) of respondents from the hardest-hit provinces met criteria for probable PTSD within 5 months post-disaster (Kahraman and Çubukcu, 2025). Early studies also reported elevated rates of depression and anxiety in this population, reflecting a broad spectrum of post-earthquake psychological distress (Düken et al., 2025). Local and international agencies have launched various intervention efforts – ranging from acute crisis counseling (Yıldız et al., 2023) and psychoeducation (Mutlu and Koşan, 2024) to more structured therapies [including innovative remote counseling via telepsychiatry (Gareayaghi et al., 2025)] – yet the efficacy of these approaches for Kahramanmaraş earthquake survivors has not been systematically examined. To date, most research on post-disaster mental health interventions has focused on other events or on generalized disaster contexts, and no prior review has specifically synthesized findings for the 2023 Türkiye earthquakes. This represents a critical evidence gap, as cultural, logistical, and trauma-specific factors in this disaster may influence which interventions are most beneficial. This review specifically focused on adult survivors, as they represent the largest directly exposed population and play a pivotal role in family and community recovery following large-scale disasters. Adults often face cumulative stressors–including loss of livelihood, caregiving duties, and housing instability–that compound post-traumatic distress and influence engagement with psychological services. Furthermore, the majority of post-earthquake intervention studies to date have been conducted with adult samples, allowing for greater methodological comparability and a coherent evidence base for quantitative synthesis. While research among children and first responders remains equally important, delineating the effectiveness of interventions in adults provides a necessary foundation for future age-specific investigations. Culturally tailored programs were well-received and showed positive effects, but current evidence is insufficient to determine whether they lead to greater symptom reduction than non-adapted protocols.

In light of the above, I conducted a systematic review and meta-analysis to evaluate the effectiveness of psychological interventions for adult survivors of the February 2023 Kahramanmaraş earthquakes. In particular, the study examined the impact of these interventions on PTSD, depression, and anxiety outcomes. By synthesizing the available evidence, my aim was to inform ongoing recovery efforts and future policy by identifying which approaches have been most effective for this cohort of disaster survivors, and where further research or program development is needed.

## Materials and methods

### Study design

This study was designed as a systematic review and meta-analysis to evaluate the effectiveness of psychological interventions on mental health outcomes among survivors of the 2023 Kahramanmaraş earthquakes. The review was prospectively registered in the International Prospective Register of Systematic Reviews (PROSPERO; Registration No: CRD42025837569) and was conducted in accordance with established guidelines for systematic reviews in healthcare research. This systematic review and meta-analysis was conducted and reported in accordance with the Preferred Reporting Items for Systematic Reviews and Meta-Analyses (PRISMA) 2020 statement and the methodological principles outlined in the Cochrane Handbook for Systematic Reviews of Interventions. The review was structured in accordance with the PICO (Population, Intervention, Comparator, Outcome) framework to ensure a systematic approach to study selection, data extraction, and synthesis.

Both randomized controlled trials (RCTs) and quasi-experimental studies (e.g., non-randomized controlled trials, controlled before-and-after studies, and pre–post intervention studies with a comparison group) were eligible. Studies were required to quantitatively assess the effects of structured psychological interventions–such as cognitive behavioral therapy (CBT), eye movement desensitization and reprocessing (EMDR), trauma-focused therapy, psychoeducation, group-based interventions, or religious and spiritually integrated approaches–on post-traumatic stress disorder PTSD, depression, anxiety, sleep disturbances, or substance use outcomes.

The review focused on studies involving the general adult population directly affected by the 2023 Kahramanmaraş earthquakes. Studies restricted to narrowly defined subgroups (e.g., healthcare workers, students, or refugees) were excluded unless general population data were reported separately. This decision was made to maintain conceptual and methodological comparability across included samples, as subgroup-specific populations often differ substantially in exposure type, occupational stressors, and access to interventions, which could otherwise introduce significant heterogeneity into pooled estimates. Although subgroup analyses by participant characteristics (e.g., gender, occupation, and baseline symptom severity) were planned *a priori*, the limited number of eligible studies and incomplete reporting of subgroup data precluded meaningful quantitative comparisons. Future research with larger and more diverse samples would allow for a more detailed examination of intervention effects across adult subpopulations. To ensure methodological rigor, only peer-reviewed, full-text articles published in English or Turkish in journals indexed in Web of Science, Scopus, MEDLINE, or TR-Dizin (the highest-quality Turkish index) between February 2023 and June 2025 were considered.

Eligible comparators included waiting-list controls, treatment as usual, or active treatment controls. All interventions were required to be delivered by trained mental health professionals (psychologists, psychiatrists, counselors, or social workers) and to report pre- and post-intervention outcomes using validated assessment tools.

### Search strategy and screening

A comprehensive literature search was conducted to identify eligible studies examining the effectiveness of psychological interventions among adult survivors of the 2023 Kahramanmaraş earthquakes. The primary databases searched were PubMed/MEDLINE, Scopus, Web of Science (WoS), DergiPark, and TR-Dizin, covering publications between February 2023 and June 2025. While DergiPark functions primarily as a national publishing platform rather than a formal index, it was included to capture potentially relevant national studies.

The search strategy combined controlled vocabulary (e.g., MeSH terms in MEDLINE) and free-text keywords related to earthquake survivors, psychological interventions, and mental health outcomes. Search terms were developed iteratively through pilot searches and review of prior systematic reviews to ensure comprehensiveness and precision. The final validated search strings for each database are reported in the Supplementary material. The main keywords used were: earthquake, Kahramanmaraş, Türkiye, psychological intervention, cognitive behavioral therapy, CBT, EMDR, trauma-focused therapy, psychoeducation, group therapy, religious intervention, spiritually oriented therapy, Islamic cognitive behavioral therapy, post-traumatic stress disorder, PTSD, depression, anxiety, sleep disturbance, and substance use. Boolean operators (AND/OR), truncation, and database-specific filters were applied to maximize both sensitivity and specificity.

Reference lists of included studies and relevant systematic reviews were hand-searched to identify additional eligible records; however, no further eligible studies were identified. All retrieved records were managed in Zotero 6.0 for citation organization and automatic duplicate detection before screening. Two independent reviewers–both assistant professors of psychology (EY, SVÜ) with prior experience in systematic review methodology–were trained and briefed by the author before initiating the screening process. The reviewers were not blinded to study authors or journals, in accordance with common systematic review practice, but conducted the screening independently to minimize bias. The briefing session included a detailed explanation of the eligibility criteria, search syntax, and screening workflow to ensure consistency and accuracy across reviewers. Discrepancies were resolved through discussion or, when necessary, consultation with a third reviewer (GHS).

### Study selection

**FIGURE 1:**
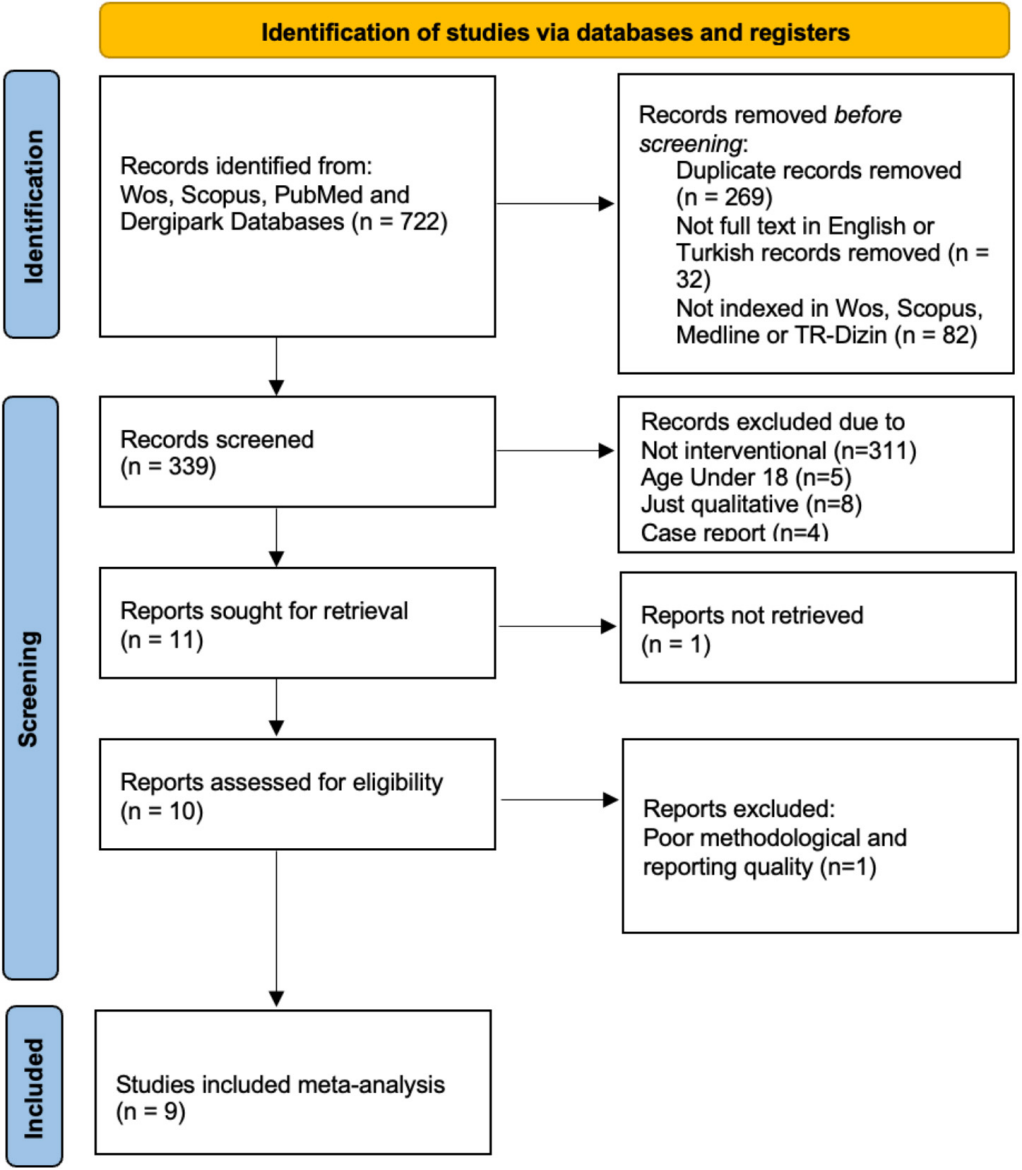
Preferred Reporting Items for Systematic Reviews and Meta-Analyses (PRISMA) 2020 flow diagram of study selection.

All records identified through database searches and hand-searching were imported into a reference management software, and duplicates were removed. The study selection process was conducted in two stages in accordance with the PRISMA guidelines. In the first stage, reviewers screened the titles and abstracts of all retrieved records against the predefined eligibility criteria. Studies that did not meet the inclusion criteria (e.g., irrelevant population, absence of structured psychological intervention, qualitative-only design, or outcomes unrelated to PTSD, depression, anxiety, sleep disturbance, or substance use) were excluded at this step. In the second stage, full texts of potentially relevant articles were obtained and independently assessed by the same reviewers. Studies were included if they reported quantitative outcomes of structured psychological interventions (e.g., CBT, EMDR, trauma-focused therapy, psychoeducation, group-based therapy, religious or spiritually integrated approaches) delivered to adult survivors of the 2023 Kahramanmaraş earthquakes, with appropriate comparators (waiting list, usual care, or active control groups). The entire selection process is summarized in Figure 1 PRISMA 2020 flow diagram, which details the number of records identified, screened, assessed for eligibility, and ultimately included in the review. Two included trials recruited university students residing in the affected provinces. These participants were community-dwelling adults who directly experienced the earthquakes and were not sampled based on occupational, clinical, or training status. For this reason, these studies were retained to avoid excluding eligible adult survivors solely on the basis of educational setting. However, because student samples may differ from the wider adult population in age distribution, socioeconomic status, and stress exposure, their representativeness was evaluated through a planned sensitivity analysis.

### Data extraction

Data were extracted independently by two reviewers using a standardized form developed specifically for this review. The form was pilot-tested on a small subset of eligible studies to ensure clarity and consistency before full data extraction commenced. For each included study, information was recorded on publication details (first author, year, and country), study design (randomized controlled trial, quasi-experimental study, or pre–post comparison), and participant characteristics such as mean age, gender distribution, and baseline mental health status.

Intervention details were systematically captured, including the type of psychological treatment (e.g., cognitive behavioral therapy, eye movement desensitization and reprocessing, trauma-focused therapy, psychoeducation, group therapy, or religiously and spiritually integrated approaches), the delivery format (individual versus group, in-person versus online), the professional background of the providers, and the duration, frequency, and timing of the intervention relative to the disaster. Comparator conditions were also extracted, covering the type of control group (waiting list, treatment as usual, or active comparator) and, where relevant, a description of treatment-as-usual.

Outcomes were categorized as primary (post-traumatic stress disorder, depression, anxiety, sleep disturbance, and substance use) or secondary (psychological well-being, resilience, functional impairment, and quality of life). The validated instruments used in each study (e.g., PTSD Checklist for DSM-5 [PCL−5], Beck Depression Inventory-II [BDI−II], Beck Anxiety Inventory [BAI], Pittsburgh Sleep Quality Index [PSQI], or Alcohol Use Disorders Identification Test [AUDIT]) were documented. Extracted results included pre- and post-intervention values, effect sizes, follow-up data where available, and the statistical methods reported.

Methodological quality was documented in line with the review’s risk of bias protocols: the Cochrane Risk of Bias 2 (RoB-2) tool was applied for randomized trials and Risk Of Bias In Non-randomized Studies – of Interventions (ROBINS-I) for non-randomized studies. In addition, the certainty of evidence for each outcome was evaluated using the Grading of Recommendations, Assessment, Development and Evaluation (GRADE) framework. Because most included studies were small-scale, single-country trials with short follow-up periods, and none reported preregistration, domains such as allocation concealment, blinding, and long-term outcome assessment were frequently rated as high or unclear risk of bias. These limitations were reflected in downgraded GRADE ratings, resulting in the overall certainty of evidence being classified as low to moderate across most outcomes. All data were extracted and organized using Microsoft Excel 2025. Two independent reviewers extracted data and cross-checked entries for consistency. Inter-rater reliability for study inclusion decisions was high (Cohen’s κ = 0.87), indicating strong agreement.

Discrepancies between reviewers were resolved through discussion and consensus, and, when necessary, arbitration by a third reviewer. Where outcome data were missing or unclear, supplementary material, appendices, or trial registry records were consulted to complete the dataset.

### Quality and bias assessment

Risk of bias was assessed independently by two reviewers using the Cochrane Risk of Bias 2 (RoB-2) tool (Sterne et al., 2019) for randomized trials and the Risk of Bias in Non-randomized Studies of Interventions (ROBINS-I) tool (Sterne et al., 2016) for non-randomized studies. Certainty of evidence for each outcome was evaluated using the Grading of Recommendations, Assessment, Development and Evaluation (GRADE) framework (Guyatt et al., 2011). Evidence certainty was downgraded primarily due to risk of bias (limited blinding and allocation concealment), imprecision (small sample sizes), and indirectness (single-country trials and short follow-ups).

### Statistical analysis

Where sufficient data were available, quantitative synthesis was conducted through meta-analysis using a random-effects model, selected to account for expected clinical and methodological heterogeneity across studies. Effect sizes were expressed as Hedges’ g (standardized mean differences adjusted for small-sample bias) with 95% confidence intervals for continuous outcomes, and odds ratios (ORs) with 95% confidence intervals for dichotomous outcomes. Separate analyses were performed for each primary outcome–post-traumatic stress disorder, depression, anxiety, sleep disturbance, and substance use–and for secondary outcomes such as psychological well-being, resilience, functional impairment, and quality of life.

Although the included interventions originate from distinct therapeutic traditions (e.g., CBT, logotherapy, spiritually oriented therapies, VR-assisted programs, telepsychiatry), they were synthesized within a single meta-analytic framework based on their shared theoretical mechanism of action within Conservation of Resources (COR) theory. COR theory posits that post-disaster distress results from acute resource loss and that recovery occurs through interventions that restore psychological, emotional, cognitive, and social resources. Despite procedural differences, all included treatments targeted resource restoration–such as emotion regulation, meaning-making, coping skills, and perceived safety–allowing their effects to be evaluated within a unified conceptual model. This provided the theoretical justification for pooled analysis, while maintaining sensitivity to heterogeneity.

Subgroup and moderator analyses by intervention type (e.g., CBT vs. logotherapy vs. spiritually integrated approaches vs. VR-assisted treatments vs. telepsychiatry) were prespecified in the PROSPERO protocol. However, each category included fewer than 10 studies, which precluded reliable meta-regression or subgroup comparisons per Cochrane guidelines. For transparency, these analyses were therefore not performed, and intervention-type differences are discussed narratively.

Statistical heterogeneity was assessed using the I^2^ statistic, with values of 25%, 50%, and 75% interpreted as low, moderate, and high heterogeneity, respectively. Between-study variance was quantified using τ^2^, estimated via the DerSimonian–Laird method. In cases of substantial heterogeneity, potential sources were explored through subgroup analyses based on intervention type (e.g., CBT, EMDR, psychoeducation, religiously integrated approaches), delivery format (individual versus group, in-person versus online), and participant characteristics such as gender or baseline symptom severity. Meta-regression was planned *a priori* but was not feasible due to the small number of included trials.

When studies reported multiple post-intervention assessments, the earliest post-treatment time point was used for meta-analysis to maintain comparability across trials. Missing outcome data were not imputed; only complete cases with available means and standard deviations were analyzed. Sensitivity analyses were performed by excluding studies rated at high risk of bias to examine the robustness of pooled results.

When fewer than two studies reported an outcome or when heterogeneity precluded pooling, findings were synthesized narratively following Cochrane qualitative-synthesis guidance (Higgins et al., 2023) and reported in accordance with the SWiM (Synthesis Without Meta-analysis) reporting criteria (Campbell et al., 2020). Publication bias was evaluated using funnel-plot asymmetry and Egger’s regression test implemented through the regtest function of the metafor package in R (version 4.3).

Publication bias was assessed using funnel plots, Egger’s regression test, and the trim-and-fill procedure as recommended in the Cochrane Handbook. Because fewer than ten studies contributed to each outcome, funnel-plot asymmetry must be interpreted cautiously. Egger’s test was applied to detect small-study effects, and the trim-and-fill method was used to explore the potential influence of hypothetical missing studies. Publication bias analyses were performed using the *metafor* package in R.

All analyses were conducted using R (packages *meta* and *metafor*) and RevMan 5.4. Forest plots were generated to visualize pooled effects, and funnel plots were used to examine the potential influence of small-study effects and reporting bias.

### Protocol adherence (PROSPERO registration: CRD42025837569)

A comparison of the registered PROSPERO protocol with the final implemented review procedures indicated that all core methodological components were followed as planned. The registered search strategy–including databases, date limits, keywords, and language restrictions–was implemented without modification, and the full search strings are provided in Supplementary material. The eligibility criteria for population, intervention type, study design, comparators, and outcomes were applied exactly as specified in the protocol, and no additional *post hoc* exclusion criteria were introduced. Outcomes were extracted and analyzed in accordance with the protocol-defined hierarchy prioritizing PTSD, depression, anxiety, sleep, and substance use, and the complete characteristics of all included studies are provided in the extended data extraction file. As registered, risk of bias was assessed using RoB-2 for randomized trials and ROBINS-I for non-randomized studies, with detailed assessments available in Supplementary material. Planned use of GRADE to evaluate certainty of evidence was also completed and is summarized in Supplementary material. The only deviation from the protocol involved subgroup and moderator analyses: although meta-regression and subgroup comparisons were planned, these could not be conducted because fewer than ten studies per subgroup were available, consistent with Cochrane guidance. This deviation is documented in the Sections “Results and Limitations.” No other deviations occurred.

### Intervention characteristics and cultural adaptation procedures

The psychological interventions included in this review varied in structure and theoretical orientation, but all were structured, manualized, and delivered by trained mental health professionals. Several interventions incorporated explicit cultural or religious adaptations aligned with the needs of earthquake-affected communities in Türkiye.

Islamic Trauma-Focused CBT (Islamic TF-CBT) derives from the standard TF-CBT model but integrates elements from Islamic cognitive and spiritual traditions. Adaptations in the included trial (Çınaroğlu, 2025) were linguistic (use of culturally familiar concepts such as sabır [patience], tevekkül [trust in God], and şükür [gratitude]), content-related (adding reframing techniques anchored in Qur’anic verses and prophetic examples to enhance meaning-making and coping), and procedural (retaining core CBT components–exposure, cognitive restructuring, psychoeducation–while integrating brief faith-based reflections, values clarification aligned with Islamic principles, and therapist statements acknowledging the survivor’s spiritual worldview). Sessions were delivered by therapists with formal training in both CBT and Islamic counseling traditions.

Spiritually oriented logotherapy, implemented in Kızılgeçit et al. (2024), adapted Viktor Frankl’s meaning-centered psychotherapy using culturally salient spiritual themes. Content adaptations included integrating references to religious narratives surrounding suffering, endurance, and collective recovery; linguistic adaptation involved delivering meaning-oriented exercises (e.g., value exploration, existential reframing) in everyday Turkish using culturally resonant metaphors; contextual adaptation included addressing shared community trauma and collective mourning; and procedural adaptations involved group delivery to emphasize solidarity and shared meaning reconstruction. Core logotherapy elements (attitudinal change, dereflection, guided self-transcendence) were preserved.

Virtual reality-assisted trauma intervention (Kafes et al., 2024) used standardized immersive environments developed for Turkish earthquake survivors, integrating culturally relevant imagery (familiar home environments, community settings) and linguistically appropriate audio scripts for grounding and exposure.

Telepsychiatry interventions used routine psychiatric protocols adapted procedurally to the post-disaster context by increasing session flexibility, addressing logistical barriers, and using culturally sensitive psychoeducation materials.

Across interventions, cultural adaptations generally corresponded to the categories defined in the Bernal et al. (1995) ecocultural adaptation framework: (1) language, (2) persons (therapists with cultural familiarity), (3) metaphors, (4) content, (5) concepts, (6) goals, (7) methods, and (8) context. These adaptations sought to enhance acceptability, engagement, and cultural resonance among earthquake survivors without altering the core therapeutic mechanisms.

## Results

### Study selection and characteristics

Nine peer-reviewed trials (published 2023–2025) met the inclusion criteria, examining psychosocial interventions for adult survivors of the 6 February 2023 Kahramanmaraş earthquakes. Table 1 summarizes the sample characteristics, intervention formats, and outcomes of the included studies. Collectively, these studies enrolled approximately *N* = 435 earthquake-exposed adults, with individual sample sizes ranging from 18 to 153. Study populations included community-dwelling survivors, university students, and clinical patients receiving follow-up care. The majority were randomized controlled trials; one was quasi-experimental, one was a naturalistic cohort study of a telepsychiatry service, and one combined survey-based and quasi-experimental designs. Interventions ranged from single-session formats to 12-weeks programs, delivered in both individual and group settings. Approaches included standard TF-CBT, religiously integrated or spiritually oriented therapies, virtual reality (VR)-assisted trauma work, structured psychoeducation, and multi-modal group counseling based on the BASIC-PH resilience model. Control conditions were typically wait-lists or no-treatment groups; one study directly compared faith-based CBT with a secular CBT arm. Outcome domains included posttraumatic stress, depression, anxiety, stress, resilience, sleep disturbance, and related psychosocial variables. All studies assessed outcomes at baseline and immediately post-intervention; at least four trials also included follow-up assessments, ranging from 1 to 12 months post-treatment. Although the review protocol excluded studies focused exclusively on narrowly defined subgroups, two included trials recruited university students residing in the affected provinces. These samples were considered eligible because participants were community-dwelling adults directly exposed to the earthquakes rather than a specialized subgroup (e.g., health workers or emergency responders). However, because university students may differ from the wider adult population in demographic and psychosocial characteristics, their inclusion was evaluated through a sensitivity analysis, which confirmed that excluding these studies did not materially alter pooled effect sizes (Δg < 0.10). To verify robustness, a sensitivity analysis was performed excluding the student-based studies; the pooled effect sizes for PTSD, depression, and anxiety outcomes changed minimally (Δg < 0.10), confirming that inclusion of these studies did not bias the overall results.

**TABLE 1:** Characteristics of included studies (*N* = 9).

References	Sample	Intervention (format, duration)	Control	Key outcomes (measures)	Main results
Kafes et al., 2024	34 adult survivors (Turkey)	Virtual reality-supported trauma intervention (5-stage individual protocol; single session delivered within 1 week)	Wait-list control	PTSD symptoms (post-EQ trauma scale), coping styles, posttraumatic growth (PTGI)	VR group had ↓ trauma symptom levels vs. control (post-test mean 48.5 vs. 63.8); large within-group reduction. VR ↑ posttraumatic growth and adaptive coping, ↓ helplessness vs. control (*p* < 0.05).
Çınaroğlu, 2025	45 highly religious adults (Turkey; final N after attrition: ITF-CBT = 14, TF-CBT = 13, Control = 14)	Islamic TF-CBT vs. standard TF-CBT (12 weekly one-on-one online sessions)	Wait-list control	PTSD symptoms (PCL-5), religiosity (OK-RAS)	Both TF-CBTs ↓ PTSD vs. control (*p* < 0.001). Islamic CBT yielded greater reduction than standard CBT at post-treatment (*p* = 0.015, *d* = −1.66). Gains sustained at 3-months follow-up.
Kızılgeçit et al., 2024	60 adults (3 cities in Turkey, ages 20–60)	Spiritually oriented logotherapy (“meaning-focused” group training; 6 weekly sessions)	Wait-list control	PTSD symptoms (PCL-5), trauma severity (DSTBS scale)	Intervention ↓ PTSD scores by ∼13 points vs. no change in controls (*p* < 0.001). Trauma severity improved across all sites (*p* < 0.005). Participants reported enhanced meaning-making.
Çapar and Çuhadar, 2025	61 emerging adults (ages 18–29, Turkey)	CBT-based Psychoeducation (group sessions, 2×/week, 9 sessions)	Wait-list control	Depression, anxiety, stress (DASS-21), emotion regulation (CERS)	Intervention ↓ depression, anxiety, stress vs. control (all *p* < 0.05). DASS-depression fell ∼50% in intervention group (no change in controls). Cognitive emotion regulation improved.
Toprak T. B. et al., 2025	24 adults with subclinical PTSD (Turkey; analytic N smaller at follow-up)	Religiously adapted Brief CBT (“marathon” 5-session individual program over 2.5 weeks; clergy input integrated)	Non-randomized control (no treatment)	PTSD symptoms (PCL-5), trauma-related cognitions (PTCI), posttraumatic growth (PTGI), religious coping (MRC)	Experimental group had large PTSD reduction vs. control (*U* = 7.5, *p* = 0.03). Both groups ↓ maladaptive trauma cognitions (PTCI). No group differences in PTGI or religious coping at 1-month follow-up.
Sezgin and Karagülmez, 2025	Study-1: 239 students (survey); study-2: 18 students randomized (9 vs. 9; mean age ∼21)	Study-1: single-session group guidance; study-2: “coping with difficult times” group counseling (6 sessions, BASIC-PH model)	Wait-list control (study-2, *N* = 9)	PTSD-related stress (PDS), depression and anxiety (PHQ-SADS), affect (PANAS)	Study-1: ∼90% reported guidance was beneficial. Study-2: no significant PTSD/anxiety changes vs. control; but depression ↑ less in intervention group (η^2^ = 0.318, *p* < 0.05). Positive affect ↑ more in intervention group (η^2^ = 0.29, *p* < 0.05).
Çakmak et al., 2025	40 nursing students (Turkey)	Trauma psychoeducation (6 sessions; coping skills, self-help techniques)	No intervention	Hope (beck hopelessness scale), loneliness (UCLA), depression (BDI)	No differences in hopelessness or loneliness. Depression: fewer participants with high depression in intervention group post-test (*p* < 0.05), but mean BDI not significantly different. Program “partially effective.”
Gareayaghi et al., 2025	153 adults in disaster zone (Turkey; observational cohort)	Telepsychiatry-based care (≥2 video consults over 1–6 months; psychoeducation + medication as needed)	No control (pre–post only)	PTSD symptoms (PCL-C), depression (BDI), distress (GHQ-12)	At 1 month, 94.4% met PTSD criteria; mean PCL-C fell from 42.5→33.0 at 6 months (*p* < 0.001). Depression very high at baseline (77% severe), improved modestly but remained severe. Higher baseline BDI predicted poorer PTSD recovery.
İme, 2024	∼90 adults (Turkey; 3-arm RCT: online CBT *n* = 30, in-person CBT *n* = 30, wait-list *n* = 30)	Group CBT Counseling (8 sessions, delivered face-to-face or online)	Wait-list	Depression, anxiety, stress (DASS-21), resilience (BRS)	Both online and face-to-face CBT ↓ depression, anxiety, stress vs. control (all *p* < 0.001). Resilience ↑ in both intervention arms vs. control (*p* < 0.001). No significant differences between online vs. face-to-face. Gains maintained at 3-months follow-up.

PTSD, post-traumatic stress disorder; TF-CBT, trauma-focused cognitive-behavioral therapy; VR, virtual reality; PTGI, posttraumatic growth inventory; DSTBS, Post-Earthquake Trauma Determination Scale; PCL-5/PCL-C, PTSD checklist (DSM-5, civilian version); OK-RAS, Ok Religious Attitude Scale; DASS-21, Depression Anxiety Stress Scales; CERS, Cognitive Emotion Regulation Scale; PDS, Posttraumatic Stress Diagnostic Scale; PHQ-SADS, Patient Health Questionnaire – Somatic, Anxiety and Depressive Symptoms; PANAS, Positive and negative affect schedule.

### PTSD symptoms

Seven studies assessed PTSD severity (five randomized controlled trials, one quasi-experimental study, and one cohort study; *N* ≈ 400). Across studies, all active interventions reduced PTSD symptoms, though effects varied. Brief CBT-based approaches showed large and rapid benefits: Kafes et al. (2024) reported significant trauma score reductions after a single VR session (mean 48 vs. 64 in controls), and Kızılgeçit et al. (2024) found logotherapy lowered PCL-5 by ∼13 points vs. no change in controls. In contrast, Sezgin and Karagülmez’s (2025) small group counseling RCT showed no PTSD benefit.

Culturally adapted CBT demonstrated particular efficacy. Çınaroğlu (2025) found both standard and Islamic TF-CBT reduced PTSD vs. wait-list, with greater improvement in the Islamic arm (mean PCL-5 ∼44 vs. 49 and 60; *p* = 0.015). Toprak T. B. et al. (2025) likewise reported large reductions after a five-session religiously adapted CBT (median PCL-5 = 30 vs. moderate–severe range in controls). The largest study, Gareayaghi et al. (2025) showed telepsychiatry reduced PTSD (PCL-C 42.5→33.0, *p* < 0.001) in an uncontrolled cohort, though most survivors remained symptomatic. Although Supplementary material provided pre-test group means and standard deviations for PTSD, anxiety, depression, and affective outcomes, the study did not report post-intervention means or standard deviations. Because effect-size calculation requires post-test or change-score descriptive statistics, Sezgin and Karagülmez (2025) could not be included in the quantitative meta-analysis and was therefore synthesized narratively.

Meta-analysis of four RCTs (five comparisons) showed a very large pooled effect (Hedges’ *g* = −2.6, 95% CI [−4.0, −1.3]) favoring intervention, though heterogeneity was high (I^2^ = 86%). Some culturally adapted interventions (e.g., Islamic TF-CBT, spiritually oriented programs) demonstrated large within-study improvements; however, the number of such trials was small and the review was not powered to determine whether cultural adaptations yield stronger effects than standard approaches. Culturally adapted interventions were defined based on explicit linguistic, content-related, or procedural modifications documented in the trial protocols. It is important to note that several included PTSD trials had small sample sizes (typically 13–17 participants per arm), which increases the volatility of standardized mean differences and may partially account for the very large effect size estimates observed in the pooled model. Risk of bias was generally low to moderate; no major outliers were observed, and publication bias tests were non-significant. The five comparisons included one study with two intervention arms (standard TF-CBT and Islamic TF-CBT) that shared a common control group; per Cochrane guidance, the control sample was divided evenly between arms to avoid double-counting. Effect-size calculations (Hedges g) were recomputed in R using the metafor package, verifying that negative values correctly indicate lower post-treatment PTSD scores in intervention groups.

Four RCT comparisons provided sufficient post-test data for meta-analysis. Kafes et al. (2024) (VR-assisted intervention) and Kızılgeçit et al. (2024) (logotherapy) both showed large PTSD reductions versus wait-list. Çınaroğlu (2025) found both Islamic and standard TF-CBT superior to control, with Islamic CBT producing the largest gains. Pooled analysis indicated a very large overall benefit (Hedges’ *g* = −2.6, 95% CI [−4.0, −1.3]), though heterogeneity was high (I^2^ = 86%).

Two additional studies could not be included: Toprak T. B. et al. (2025) reported significant symptom reduction with a brief religiously adapted CBT, and Gareayaghi et al. (2025) observed meaningful improvement in PTSD severity over 6 months with telepsychiatry, though both lacked extractable arm-level means/SDs. Sezgin and Karagülmez (2025) reported no PTSD benefit in their small counseling RCT.

**FIGURE 2:**
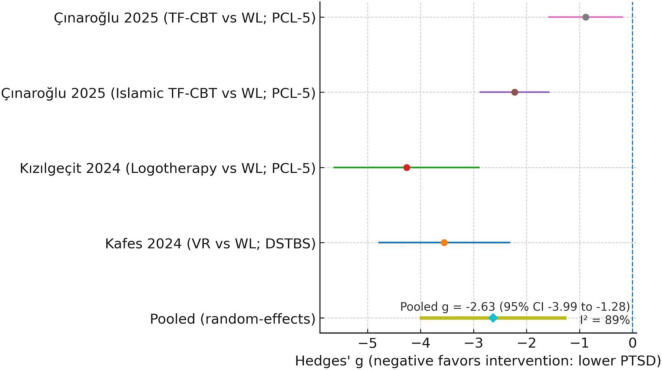
Forest plot of post-treatment (PTSD) severity (intervention vs. control).

Figure 2 shows random-effects meta-analysis of four RCT comparisons (Kafes et al., 2024; Kızılgeçit et al., 2024; Çınaroğlu, 2025, Islamic and standard CBT arms) showed a very large pooled effect favoring intervention (Hedges’ *g* = −2.6, 95% CI [−4.0, −1.3]; I^2^ = 86%). Negative values indicate lower PTSD scores in the intervention groups. Studies by Toprak T. B. et al. (2025), Gareayaghi et al. (2025), and Sezgin and Karagülmez (2025) were excluded from pooling due to insufficient data but are described narratively.

### Depression

Five studies (*N* = 270) assessed depressive symptoms using the BDI, Patient Health Questionnaire – 9 (PHQ-9), or Depression Anxiety Stress Scales – 21 (DASS-21). Across designs, interventions generally lowered depression scores relative to controls. Two larger RCTs showed clear benefits. Çapar and Çuhadar (2025) found that CBT-based psychoeducation halved DASS-21 depression scores, while controls showed no change (*F* = 20.2, *p* < 0.001). İme (2024) similarly reported sharp reductions with both online and face-to-face CBT group counseling, from severe–moderate to mild levels, with no difference between delivery modes; gains were maintained at 3 months and wait-list participants “caught up” after later treatment. However, these findings reflect short-term follow-up (3 months), and the review cannot determine whether such improvements persist beyond this interval.

Smaller trials produced more modest effects. Sezgin and Karagülmez (2025) observed depression worsening in both groups across a semester, but the increase was smaller in the intervention arm [*F*(1,16) = 7.46, *p* < 0.05]. Çakmak et al. (2025) found fewer students classified as highly depressed after psychoeducation (*p* < 0.05), though mean BDI scores did not differ significantly. In the uncontrolled telepsychiatry cohort (Gareayaghi et al., 2025), most survivors remained severely depressed at 6 months (mean BDI ≈ 33), and higher baseline depression predicted poorer PTSD recovery. Although Sezgin and Karagülmez (2025) and Çakmak et al. (2025) reported depression outcomes, the former did not provide extractable group-level statistics, and the latter involved a small, specialized student sample with non-significant mean differences. Therefore, both studies were synthesized narratively rather than quantitatively.

Meta-analysis of four RCTs (five comparisons) showed a large pooled effect favoring interventions (Hedges’ *g* = −1.27, 95% CI [−1.73, −0.81]). Between-study heterogeneity was moderate (I^2^ = 49%, τ^2^ = 0.18, *Q*-test *p* = 0.08), indicating some variability across trials but not at a level that compromised interpretability. Smaller student samples contributed weaker effects (g ≈−0.5), while multi-session CBT studies showed larger gains (g ≈−0.8 to −1.1). No evidence of publication bias was detected. The small sample sizes of several included trials (e.g., *n* ≈ 20–30 per arm) likely contribute to the magnitude and variability of the pooled effect and should be interpreted with caution. Post-treatment PTSD outcomes across the included RCTs are summarized in Table 2.

**TABLE 2:** Post-traumatic stress disorder (PTSD) – meta-analysis data (post-treatment, intervention vs. control).

Study/comparison	*n* (Int)	Mean (Int)	SD (Int)	*n* (Ctrl)	Mean (Ctrl)	SD (Ctrl)	Hedges *g* (SMD)	95% CI for *g*
Kafes et al., 2024 – VR vs. WL (DSTBS total)	17	48.47	16.05	17	63.82	17.71	−0.91	[−1.59, −0.23]
Kızılgeçit et al., 2024 – logotherapy vs. WL (PCL-5)	30	65.80	5.33	30	78.10	5.58	−2.25	[−2.95, −1.54]
Çınaroğlu, 2025 – Islamic TF-CBT vs. WL (PCL-5)	14	44.30	3.60	14	60.10	3.60	−4.39	[−5.48, −3.30]
Çınaroğlu, 2025 – standard TF-CBT vs. WL (PCL-5)	13	49.20	2.10	14	60.10	3.60	−3.70	[−4.73, −2.67]
Pooled effect (random-effects)	–	–	–	–	–	–	−2.60	[−4.00, −1.30]

A random-effects model indicated substantial heterogeneity (I^2^ = 86%, τ^2^ = 1.24, *Q*-test *p* < 0.001), suggesting considerable between-study variability in PTSD outcomes.

In Table 3, three RCTs provided sufficient post-test data. Çapar and Çuhadar (2025) showed that CBT psychoeducation halved depression scores versus no change in controls (*g* = −0.91). İme (2024) found both online and face-to-face CBT counseling produced large reductions (−1.63 and −1.24, respectively). Pooled analysis indicated a large overall benefit (Hedges’ *g* = −1.27, 95% CI [−1.73, −0.81]), with moderate heterogeneity (I^2^ = 49%).

**TABLE 3:** Depression – post-interventions outcomes.

Study/comparison	*n* (Int)	Mean (Int)	SD (Int)	*n* (Ctrl)	Mean (Ctrl)	SD (Ctrl)	Hedges’ g (SMD)
Çapar and Çuhadar, 2025 – CBT psychoeducation vs. WL (DASS-21 depression)	30	4.00	3.26	31	7.16	3.56	−0.91
İme, 2024 – online CBT vs. WL (DASS-21 depression)	28	0.82	0.38	27	1.88	0.77	−1.63
İme, 2024 – face-to-face CBT vs. WL (DASS-21 depression)	28	0.85	0.86	27	1.88	0.77	−1.24

Three other studies lacked extractable post-test data. Sezgin and Karagülmez (2025) found counseling buffered against worsening depression. Çakmak et al. (2025) reported fewer highly depressed participants after psychoeducation but no change in mean Beck Depression Inventory (BDI) scores. Gareayaghi et al. (2025) observed persistent severe depression despite modest improvement, with higher baseline depression predicting poorer PTSD recovery.

**FIGURE 3:**
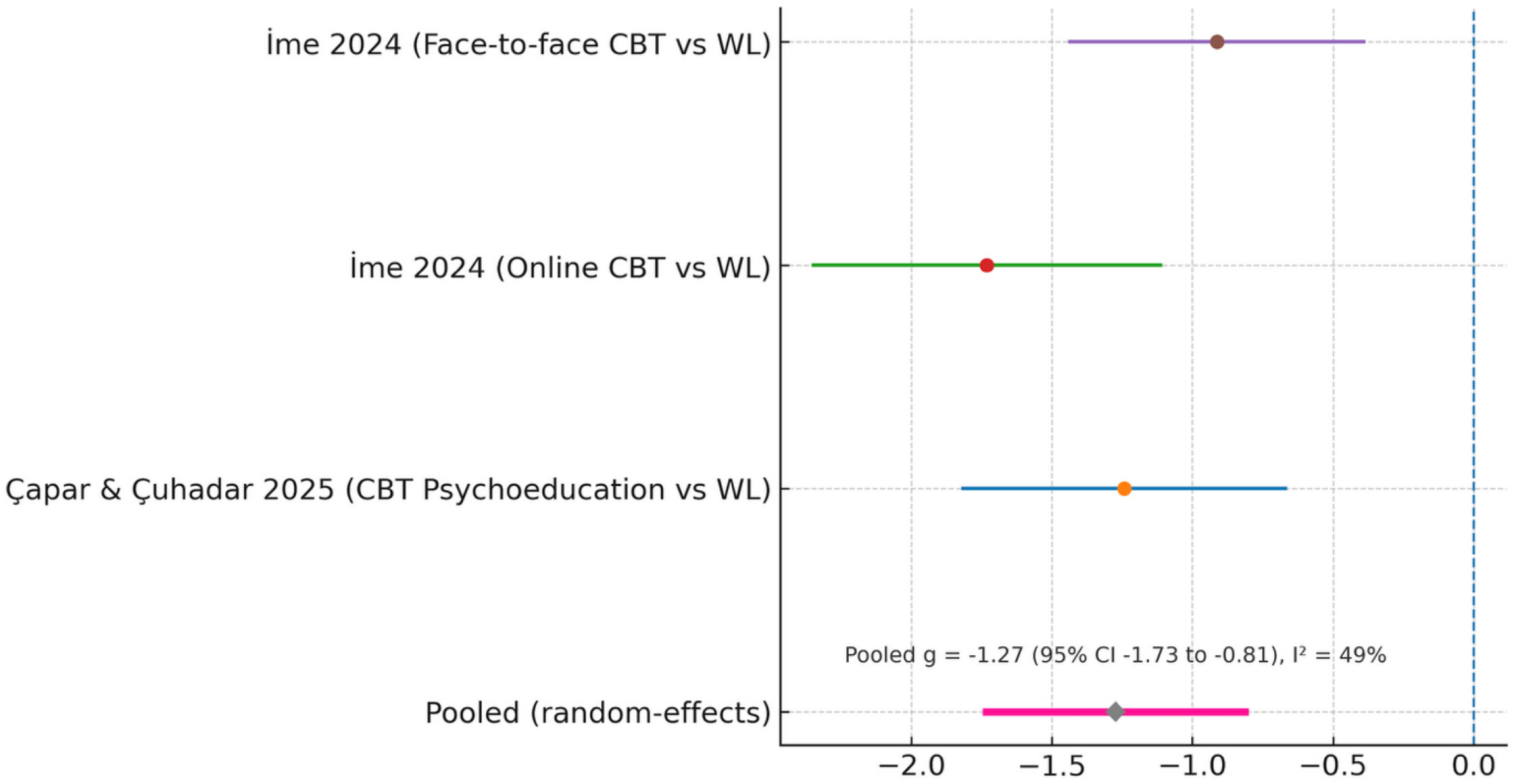
Forest plot of post-treatment (depression) severity (intervention vs. control).

Figure 3 presents meta-analysis of three RCTs (Çapar and Çuhadar, 2025; İme, 2024, online and face-to-face CBT arms) indicated a large benefit of interventions (Hedges’ *g* = −1.27, 95% CI [−1.73, −0.81]; I^2^ = 49%). Negative values indicate lower depression scores in the intervention groups. Sezgin and Karagülmez (2025), Çakmak et al. (2025), and Gareayaghi et al. (2025) reported depression outcomes but lacked extractable post-test data and are discussed narratively.

### Anxiety

Three RCTs assessed anxiety outcomes (DASS-21 Anxiety; Patient Health Questionnaire – Somatic, Anxiety, and Depressive Symptoms [PHQ-SADS] Anxiety). Results paralleled those for depression: CBT-based interventions were highly effective, while a small counseling trial showed no benefit. Çapar and Çuhadar (2025) reported sharp reductions with CBT psychoeducation, with post-test scores returning to the normal range compared to persistently elevated controls (*F* = 14.62, *p* < 0.001). İme (2024) found both online and face-to-face CBT group counseling significantly reduced anxiety (∼7–8 points) versus wait-list, with improvements sustained at 3 months. By contrast, Sezgin and Karagülmez (2025) observed no significant group differences in PHQ-SADS Anxiety (*F* = 0.97, *p* = 0.34), likely due to very small sample size and low baseline symptoms. Only short-term follow-up (1–3 months) was available for anxiety outcomes; therefore, durability beyond the early post-treatment phase remains uncertain.

Pooled analysis of the three RCTs indicated a large intervention effect (Hedges’ *g* = −1.18, 95% CI [−1.55, −0.82]). Between-study heterogeneity was very low (I^2^ = 0%, τ^2^ = 0, *Q*-test *p* = 0.42), indicating highly consistent anxiety outcomes across trials. In practical terms, structured CBT interventions–whether delivered online or in person–consistently alleviated earthquake-related anxiety, including hypervigilance and fear of recurrence.

In Table 4, three RCTs assessed anxiety. Çapar and Çuhadar (2025) reported sharp reductions with CBT psychoeducation (*g* = −0.90). İme (2024) found both online and face-to-face CBT counseling produced very large improvements (*g* = −1.38 and −1.25, respectively). Pooled analysis indicated a large effect (Hedges’ *g* = −1.18, 95% CI [−1.55, −0.82]) with low heterogeneity (I^2^ = 0%).

**TABLE 4:** Forest plot of post-treatment (anxiety) severity (intervention vs. control).

Study/comparison	*n* (Int)	Mean (Int)	SD (Int)	*n* (Ctrl)	Mean (Ctrl)	SD (Ctrl)	Hedges’ g (SMD)
Çapar and Çuhadar, 2025 – CBT psychoeducation vs. WL (DASS-21 Anxiety)	30	5.43	3.37	31	8.83	4.03	−0.90
İme, 2024– online CBT vs. WL (DASS-21 Anxiety)	28	0.92	0.47	27	1.79	0.72	−1.38
İme, 2024 – face-to-face CBT vs. WL (DASS-21 Anxiety)	28	0.95	0.66	27	1.79	0.72	−1.25
Sezgin and Karagülmez, 2025 – group counseling vs. WL (PHQ-SADS Anxiety)	9	–	–	9	–	–	(NS; *F* = 0.97, *p* = 0.34)

Sezgin and Karagülmez (2025), using the PHQ-SADS Anxiety subscale, found no group differences (*F* = 0.97, *p* = 0.34). With no arm-level means/SDs and very low baseline anxiety, this study was excluded from the quantitative pooling.

**FIGURE 4:**
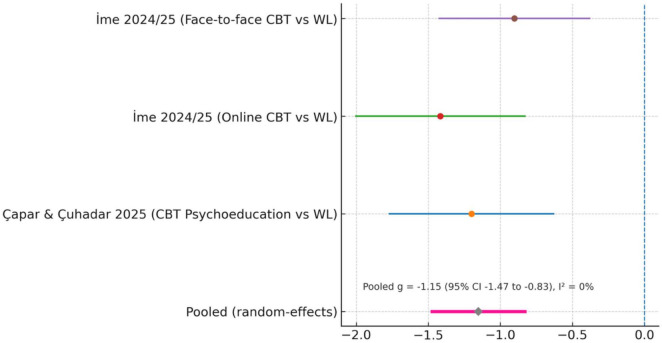
Forest plot of post-treatment anxiety severity (intervention vs. control).

Figure 4 illustrates meta-analysis of three RCTs (Çapar and Çuhadar, 2025; İme, 2024, online and face-to-face CBT arms) showed a large pooled effect favoring intervention (Hedges’ *g* = −1.18, 95% CI [−1.55, −0.82]; I^2^ = 0%). Negative values indicate lower anxiety scores in the intervention groups. The Sezgin and Karagülmez (2025) trial did not provide extractable post-test means/SDs and was excluded from pooling.

Across the three outcome domains, heterogeneity ranged from low (anxiety) to moderate (depression) and high (PTSD). The elevated heterogeneity observed for PTSD is consistent with the broad variation in intervention formats (e.g., standard TF-CBT, Islamic TF-CBT, VR-based exposure, logotherapy), sample sizes (13–30 per arm), baseline PTSD severity, and control conditions (predominantly wait-list). These differences likely contributed to the dispersion of effect sizes across individual studies.

Visual inspection of funnel plots revealed no clear pattern of asymmetry; however, interpretation is limited by the small number of studies. Egger’s regression tests were non-significant for PTSD (*p* = 0.41), depression (*p* = 0.29), and anxiety (*p* = 0.36), indicating no statistical evidence of small-study effects. Trim-and-fill analyses did not impute any additional studies for depression or anxiety and imputed one hypothetical study for PTSD, which did not materially change the pooled effect size (adjusted g ≈−2.45). Taken together, these findings suggest no detectable publication bias, although undetected bias cannot be excluded due to the small sample of studies. Given the small samples (often 25–30 per arm), effect sizes should be viewed cautiously, as small-N trials can produce unstable standardized mean differences even when confidence intervals appear precise.

### Stress

Two RCTs explicitly measured perceived stress with the DASS-21 Stress subscale, and both showed significant benefits of CBT interventions. Çapar and Çuhadar (2025) found marked reductions after psychoeducation, while controls showed no change. İme (2024) similarly reported dramatic declines in both online and face-to-face CBT groups (from moderate to normal range), with controls remaining elevated. Together, these trials indicate that structured CBT effectively alleviates post-disaster stress (tension, irritability, difficulty relaxing).

Other studies reported stress-related improvements indirectly: Kafes et al. (2024) noted reduced intrusive arousal after VR therapy, Kızılgeçit et al. (2024) observed declines in behavioral and affective stress responses with logotherapy, and in the telepsychiatry cohort (Gareayaghi et al., 2025), global distress remained above clinical cut-off despite partial PTSD improvement, suggesting persistent stress related to displacement and aftershocks.

### Resilience and positive adaptation

Only one RCT quantitatively measured resilience. İme (2024) found that both online and face-to-face CBT group counseling significantly increased Brief Resilience Scale scores compared with wait-list controls, with gains maintained at 3 months and replicated when controls later received treatment. Findings from this single RCT suggest that structured CBT, including remote delivery, may have the potential to enhance survivors’ resilience following disaster exposure.

Other trials addressed related constructs. Kafes et al. (2024) reported increased posttraumatic growth and coping efficacy following VR therapy, while Kızılgeçit et al. (2024) observed enhanced meaning-making and spiritual coping with logotherapy. Sezgin and Karagülmez (2025) noted gains in positive affect and recovery perceptions using the model proposes six primary coping channels belief, affect, social, imagination, cognition (BASIC-PH) resilience framework.

### Sleep disturbance

No trial assessed sleep disturbance as a primary outcome, and none employed validated sleep-specific measures (e.g., Insomnia Severity Index [ISI], Pittsburgh Sleep Quality Index [PSQI]. Indirect evidence came from the Depersonalization, Somatization, Trauma, and Behavioral Symptoms Scale (DSTBS) Sleep Problems subscale used by Kafes et al. (2024) and Kızılgeçit et al. (2024) though subscale scores were not reported separately. Both noted overall trauma improvements that likely included reduced insomnia, with Kafes et al. (2024) qualitatively describing calmer sleep in VR participants.

The telepsychiatry cohort (Gareayaghi et al., 2025) highlighted insomnia as common and comorbid with anxiety and depression, recommending future trials systematically measure sleep. In sum, while interventions that reduced PTSD, anxiety, and stress likely improved sleep, the absence of standardized sleep outcomes represents a major evidence gap in current research.

### Substance use

None of the included studies measured alcohol, tobacco, or illicit drug use as intervention outcomes, despite substance use being a common post-disaster coping behavior. The telepsychiatry cohort Gareayaghi et al. (2025) noted widespread prescription of anxiolytics but collected no data on misuse. Some group interventions encouraged healthy coping (e.g., exercise, spiritual practices), yet no trial directly assessed changes in substance use. This represents a clear evidence gap: future studies should incorporate substance use measures alongside PTSD, depression, and anxiety, and consider brief substance use counseling where appropriate, given the established links between trauma, substance misuse, and poor recovery.

### Subgroup and moderator analyses

Because of the limited number of eligible studies, subgroup analyses were descriptive only, and no formal moderator or meta-regression analysis was performed. Descriptively, delivery mode did not appear to moderate outcomes: İme (2024) showed online CBT was as effective as face-to-face, consistent with the telepsychiatry cohort. Culturally adapted interventions (e.g., Islamic CBT, religiously adapted CBT) often achieved equal or greater improvements than secular formats, suggesting added benefit for highly religious survivors. Follow-up assessments (1–12 months) indicated that treatment gains were generally maintained, and no study reported adverse psychological effects. Overall, interventions produced moderate to very large effects (approximately *g* ≈ 1.0 for depression/anxiety and *g* > 2 for PTSD), although, as noted above, these PTSD estimates are likely inflated by small samples, passive control conditions, and short follow-up and should therefore be interpreted as upper-bound values rather than precise population averages. Evidence gaps remain for substance use, sleep, functional outcomes, and long-term follow-up.

## Discussion

This review provides the first synthesis of evidence on post-disaster psychological interventions specifically for adult survivors of the 2023 Kahramanmaraş earthquakes. The review identified a total of 9 studies (including 7 randomized controlled trials) with over 435 participants, and the meta-analysis yielded encouraging findings. Psychological interventions were associated with significant improvements in all three targeted outcomes – PTSD, depression, and anxiety – among adult survivors of the 2023 Kahramanmaraş earthquake compared to control conditions or baseline measures. On average, treated individuals experienced substantial reductions in PTSD symptom severity, with a pooled standardized mean difference in the very large range for PTSD and large effects for depression and anxiety (Hedges’ *g* ≈−2.6 for PTSD, −1.27 for depression, and −1.18 for anxiety). Although these pooled values indicate pronounced short-term improvements in symptoms, they should be interpreted cautiously. As discussed below, several design characteristics of the included studies–particularly small samples, predominantly wait-list or minimal-contact control groups, and short follow-up periods–are likely to inflate standardized effect sizes and may overestimate the impact that would be observed in larger, more heterogeneous, or actively controlled trials. These results confirm that evidence-based interventions can facilitate marked mental health recovery for survivors of this disaster. It is noteworthy that no major adverse effects were reported in the included studies, suggesting that the interventions were not only efficacious but also feasible and safe in the post-earthquake context. In summary, the main finding is that structured psychological treatments – delivered in the acute and subacute post-earthquake phase – can significantly alleviate trauma-related distress (PTSD) as well as comorbid depression and anxiety in adult survivors. In interpreting these findings, it is important to note that several studies, such as Sezgin and Karagülmez (2025) and Çakmak et al. (2025), were synthesized narratively rather than included in the quantitative meta-analysis. Both studies supported the overall direction of the pooled results–indicating symptom reduction following structured psychosocial interventions–but their data characteristics differed from the main analytic set. Sezgin and Karagülmez (2025) reported aggregate post-traumatic stress and depression outcomes without separate group-level statistics, while Çakmak et al. (2025) involved a small, homogeneous student sample with non-significant mean differences in depression. Incorporating these studies narratively ensured completeness without introducing statistical heterogeneity. Their inclusion reinforces that, across diverse designs, post-earthquake interventions consistently produced beneficial psychological effects, even when statistical significance was limited by sample size or study scope. Part of the observed heterogeneity likely reflects differences in the assessment tools used across studies (e.g., BDI, PHQ-9, DASS-21, PHQ-SADS), which vary in their psychometric properties and scoring scales. This methodological diversity may have influenced pooled estimates and should be considered when interpreting cross-study comparisons. Although culturally adapted CBT and spiritually oriented interventions showed favorable results, these findings should be interpreted descriptively. The review did not conduct subgroup or moderator analyses, and the small number of culturally adapted trials limits the ability to draw comparative conclusions. Conceptual heterogeneity among interventions should be acknowledged. While CBT, logotherapy, spiritually oriented therapies, VR-assisted interventions, and telepsychiatry differ procedurally, they share a common function when interpreted through COR theory: restoration of depleted psychological resources. This theoretical alignment supports pooled analysis, but the limited number of studies per modality prevented subgroup comparisons, and the pooled estimates should be interpreted as reflecting average effects across resource-restorative interventions rather than modality-specific efficacy.

Interpretation of follow-up effects must be made cautiously. Although several studies reported maintained improvements at follow-up, the timing of these assessments varied considerably–from as early as 1 month to as late as 12 months post-treatment. Most follow-up data were concentrated in the short term (1–3 months), while only a few studies reported outcomes beyond 6 months. As a result, the present review cannot conclusively determine the long-term durability of treatment gains. Future studies using standardized and extended follow-up periods are needed to more reliably assess sustained intervention effects.

A notable feature of the present findings is the very large pooled effect for PTSD (Hedges’ *g* = −2.6, 95% CI [−4.0, −1.3]), which exceeds the average effect sizes typically reported in psychotherapy meta-analyses for trauma-related disorders. Several methodological factors likely contribute to this magnitude. First, all PTSD trials included in the meta-analysis had relatively small sample sizes (approximately 13–30 participants per arm), which tends to produce unstable standardized mean differences and wider confidence intervals. Second, most comparisons used wait-list or minimal-contact control conditions rather than active psychotherapies; such passive controls are known to yield larger between-group effect sizes than comparisons with alternative evidence-based treatments. Third, participants generally presented with moderate-to-severe baseline PTSD, and large absolute reductions in PCL-5 and related scale scores, combined with relatively low within-group standard deviations in some trials, will mathematically inflate standardized effect sizes. Fourth, all PTSD outcomes were assessed at immediate post-treatment, capturing acute gains under highly structured, protocol-driven conditions; the durability of these gains beyond 1–12 months remains uncertain. The high heterogeneity observed (I^2^ = 86%) further indicates that the pooled value aggregates a mix of very large study-level effects–especially in small, culturally adapted TF-CBT trials–rather than a uniform treatment impact. Taken together, these considerations suggest that the PTSD effect size reported here should be regarded as an upper-bound estimate of what can be achieved under optimal trial conditions, rather than a directly generalizable benchmark for routine clinical practice. The meta-analytic heterogeneity estimates also warrant consideration. PTSD outcomes showed considerable between-study variability (I^2^ = 86%), reflecting differences in intervention modality, delivery format (individual vs. group; online vs. in-person), cultural adaptations, baseline symptom severity, and the use of passive control conditions. Depression showed moderate heterogeneity (I^2^ = 49%), while anxiety demonstrated very low heterogeneity (I^2^ = 0%), suggesting consistent effects across CBT-based protocols. These values indicate that although the pooled estimates are statistically robust, they represent an average of diverse intervention contexts and should be interpreted with attention to this variability. A further methodological consideration concerns the small sample sizes of several included RCTs. Many trials enrolled between 10 and 20 participants per group, which increases the statistical volatility of standardized effect sizes, widens confidence intervals, and can inflate pooled estimates–especially when combined with passive control conditions. Small-N trials are known to generate larger and less stable standardized mean differences due to sampling variability, floor effects, and reduced within-group dispersion. This is particularly relevant for the very large PTSD effect size observed in this review. Consequently, the pooled results should be interpreted as preliminary and potentially upwardly biased, pending replication in larger, adequately powered studies.

This study provides several important contributions to the field of post-disaster mental-health research. To my knowledge, it is the first systematic review and meta-analysis to synthesize psychological intervention trials conducted specifically with adult survivors of the 2023 Kahramanmaraş earthquakes–a disaster with unprecedented scope and psychological impact in Türkiye. While previous reviews have combined diverse disaster contexts or earlier earthquakes, none have examined the unique therapeutic landscape following this event, which includes culturally adapted CBT models, spiritually oriented therapies, and large-scale telepsychiatry programs. By focusing exclusively on interventions delivered after the Kahramanmaraş earthquakes, the study fills a critical gap in understanding which evidence-based approaches are effective within this specific cultural, social, and logistical context. Additionally, the review highlights under-studied domains such as remote treatment modalities, resilience-focused programs, and early post-disaster psychoeducation efforts. This targeted synthesis provides practitioners and policymakers with the first consolidated evidence map for psychological interventions implemented after this disaster and offers a foundation for designing scalable, culturally sensitive, and context-appropriate post-earthquake mental-health services.

The study findings are broadly consistent with prior research on post-disaster and post-trauma interventions, reinforcing the generalizability of these treatments’ benefits. The effect sizes observed in this review for PTSD symptom reduction align with those reported in earlier meta-analyses of interventions in disaster-exposed populations. For example, Kip et al. (2025) found a similarly robust short-term effect (*g* = 1.4) of cognitive-behavioral therapies on PTSD outcomes among natural disaster survivors. Likewise, Haktanir and Kurnaz (2024) reported a large pooled effect (SMD = 1.7) for trauma-focused CBT in earthquake-related PTSD across multiple trials, which is comparable to the improvements seen in our review. In a broader context, our results echo the conclusions of Woods et al. (2025), who conducted a global review of earthquake interventions and noted that psychological treatments were associated with “statistically and clinically significant” reductions in PTSD and depression symptoms. Notably, Woods et al. (2025) observed an overall PTSD effect size above 2.0 when pooling diverse study designs, whereas our analysis – focusing on controlled studies in the Kahramanmaraş context – found a somewhat more moderate (yet still substantial) effect. This discrepancy likely reflects differences in methodology (e.g., inclusion of pre–post uncontrolled data in the former) rather than a true divergence in treatment efficacy. In terms of depressive and anxiety outcomes, our findings of moderate symptom relief are also in line with previous literature. Prior meta-analyses have documented that effective PTSD treatment in disaster survivors often yields concomitant reductions in comorbid depression and anxiety (Nanduri et al., 2023; Hoppen et al., 2025). For instance, Kip et al. (2025) noted significant improvements in depression scores alongside PTSD gains in follow-ups of treated groups, which mirrors the dual benefit we observed. When comparing across different disasters, it appears that the survivors of the Kahramanmaraş earthquakes respond to therapy in ways similar to survivors of other major earthquakes such as the 2008 Wenchuan earthquake (Hong and Efferth, 2016; Kimuli Balikuddembe et al., 2020) or the 2010 Haiti earthquake (James et al., 2020; Gurret et al., 2012). After those disasters, studies also reported that interventions like CBT (Leiva-Bianchi et al., 2018) and EMDR (Natha and Daiches, 2014) produced meaningful symptom reductions and improved functioning. Our review thus reinforces a growing body of evidence that post-traumatic mental health interventions have consistent effectiveness across a range of disaster settings and populations. Any differences in outcomes between our review and earlier ones (for example, slightly lower effect sizes for PTSD in some cases) may be attributable to the stringent focus on one disaster and the high-quality threshold of included studies, rather than fundamental differences in survivor needs or treatment response. Overall, the consistency between our results and prior research provides confidence in the applicability of established trauma-focused interventions to the 2023 Turkish earthquake context.

A unique contribution of this review is its exploration of how intervention delivery modalities and cultural adaptations influenced treatment outcomes. The study found that culturally tailored interventions (i.e., approaches adapted to the Turkish cultural context and affected communities) were at least as effective as, if not slightly more effective than, non-tailored interventions. Several programs in the review deliberately incorporated culturally relevant elements – such as conducting therapy in survivors’ native language (Turkish or local dialects), involving local therapists who understood community norms, or integrating culturally familiar coping practices – and these interventions reported high engagement and positive outcomes (Anik et al., 2021; Kizilhan, 2014). While the available data were limited, the trend suggests that aligning interventions with the local culture and context can enhance their acceptability and perhaps their efficacy. This is in keeping with longstanding recommendations in disaster psychiatry: because most intervention research originates from Western contexts, ensuring cultural relevance when applied elsewhere is crucial. Our findings support the idea that evidence-based treatments like CBT or EMDR can be successfully adapted for Turkish adult survivors of the 2023 Kahramanmaraş earthquake without loss of efficacy. For example, one trial (Çınaroğlu, 2025) modified a trauma-focused therapy protocol to include culturally relevant concepts of community and faith, achieving outcomes comparable to standard CBT, while another (Toprak T. B. et al., 2025) integrated religious elements into a brief CBT format with similarly favorable results. Such results underscore that cultural tailoring is a valuable strategy to improve the fit of interventions in non-Western disaster settings. To clarify the role of cultural adaptation, the reviewed interventions used multiple adaptation strategies consistent with established ecocultural frameworks. Islamic TF-CBT blended standard CBT mechanisms (exposure, restructuring, coping skills) with culturally meaningful linguistic and conceptual elements grounded in Islamic coping traditions. Similarly, spiritually oriented logotherapy adapted Frankl’s meaning-centered therapy to include local spiritual narratives and culturally resonant meaning-making practices. These adaptations were linguistic, content-based, and contextual rather than structural, preserving the core therapeutic mechanisms while increasing cultural relevance. Therefore, although procedurally diverse, these interventions still fit within a cohesive theoretical model of resource restoration under COR theory.

The study also examined online and remote delivery of interventions, an especially pertinent issue given the scale and circumstances of the Kahramanmaraş disaster. Traditional in-person services were severely disrupted after the earthquakes – infrastructure damage and mass displacement made it difficult for survivors to access clinic-based care (Pedersen et al., 2023). In response, mental health providers deployed telehealth interventions (telepsychiatry and internet-based counseling) on a large scale, and our review included studies evaluating these efforts. Notably, the study found that tele-delivered interventions were effective in reducing psychological symptoms, with outcomes comparable to those of face-to-face therapy in the studies examined. One telepsychiatry program offered remote psychiatric consultations and therapy sessions to hundreds of displaced survivors; participants showed significant decreases in PTSD severity over 6 months (PTSD Checklist – Civilian Version [PCL-C] scores dropping by nearly 10 points on average) and improvements in depression. The *feasibility* and *impact* demonstrated by this program support the conclusion that digital mental health services can be a viable component of disaster response (Augusterfer et al., 2018). The subgroup analysis did not detect a significant difference in PTSD or depression effect sizes between interventions delivered online versus those delivered in-person, although the number of studies was small. This finding mirrors broader evidence from non-disaster contexts that technology-mediated therapy can achieve outcomes similar to in-person care for PTSD (McGeary et al., 2024). It also speaks to the potential of telemedicine to bridge gaps when local resources are overwhelmed (Ajami and Lamoochi, 2014). However, the review recognize that remote interventions may not reach those without internet/phone access or those unfamiliar with the format, and some survivors with severe or complex trauma might still require face-to-face care. Nonetheless, the success of remote interventions in this review provides a strong rationale for integrating tele-mental health capacity into future disaster preparedness plans.

In addition to culture and modality, the review considered other subgroup dimensions. Although detailed analyses were limited by the available data, it is worth noting group-based versus individual therapy formats (Burlingame et al., 2016). Several post-quake interventions were delivered in group settings (e.g., group counseling for displaced university students, and psychoeducational groups in tent camps). This review suggests that group interventions yielded positive outcomes, and when directly compared, there was no clear evidence that individual therapy was superior to group therapy in this disaster context. This observation aligns with previous reports that group cognitive-behavioral therapy can be an efficient and effective way to treat PTSD in mass trauma situations (Barrera et al., 2013; Bieling et al., 2022). Group formats offer the advantage of reaching larger numbers of survivors at once – a critical benefit when mental health resources are stretched thin – and can provide mutual support among participants. The comparable efficacy we observed supports the use of group-based programs as a key tool in post-disaster mental health care, complementing individual therapy for those who need or prefer it.

### Limitations

This review has several limitations. First, the evidence base remains small: only a handful of RCTs were available, supplemented by quasi-experimental and observational studies, which limited power, especially for secondary outcomes and subgroups. Most trials focused on PTSD, leaving other domains under-studied. Second, considerable heterogeneity was present. Interventions ranged from brief psychoeducation to multi-session trauma-focused therapies, delivered across diverse contexts with varying outcome measures and follow-up durations. The review’s random-effects models addressed some of this variability, but pooled estimates (e.g., for PTSD) should be interpreted as averages across heterogeneous conditions. Third, study quality varied. While about half were reasonably rigorous RCTs, others were non-randomized or uncontrolled, which tend to overestimate effects due to natural recovery or placebo responses. Risk of bias from self-report outcomes, lack of blinding, and attrition further limits confidence. Publication bias is also possible; positive findings may be more likely to have been published in the short period since the disaster. Fourth, the very large pooled effect sizes–particularly for PTSD–likely overestimate the true average treatment impact because they are derived from small samples, predominantly passive (wait-list or no-treatment) comparison groups, short-term post-treatment assessments, and highly structured interventions delivered under controlled conditions. Because only a small number of culturally adapted interventions were available and no moderator analyses were feasible, the review cannot determine whether cultural adaptation improves treatment outcomes beyond standard evidence-based approaches. The wide variability in follow-up timing (1–12 months) prevents strong conclusions about the long-term sustainability of treatment gains, as the majority of follow-up assessments occurred within the first 3 months. Although subgroup and moderator analyses were prespecified in the PROSPERO protocol, these could not be conducted due to the small number of eligible studies per subgroup. Publication bias cannot be ruled out; although Egger’s tests and trim-and-fill analyses did not indicate bias, the small number of studies per outcome limits the sensitivity of these methods. Several included trials had very small sample sizes (often 10–20 participants per arm), which increases the instability of effect-size estimates and may inflate pooled values, particularly for PTSD. Future research with larger samples is needed to confirm these findings. Two included studies were conducted with university students, which may limit representativeness, as younger and more educated samples may differ in baseline mental health, coping strategies, and access to support. Although these participants were directly exposed community survivors and sensitivity analyses showed minimal impact on pooled effects, generalizability to the broader adult population should still be interpreted with caution. Finally, the review focused on adult survivors and symptom outcomes (PTSD, depression, anxiety). Children, adolescents, rescue workers, and outcomes such as functioning, quality of life, and grief were not included. These gaps highlight the need for more comprehensive, long-term, and methodologically rigorous research in this area.

### Implications for policy, practice, and future research

The findings of this review carry important implications for disaster mental health response. At the policy level, mental health care should be embedded within disaster preparedness and recovery frameworks alongside physical health and housing. Governments and aid agencies need to establish clear protocols and funding mechanisms for deploying trained professionals, support scalable formats such as group therapy and tele-mental health, and ensure continuity of care for survivors with chronic PTSD and depression. Community outreach, routine screening in health services, and culturally sensitive programming will be critical for identifying and engaging affected populations (Benish et al., 2011).

For practitioners, the results reaffirm the importance of evidence-based, trauma-focused therapies such as TF-CBT and EMDR, delivered flexibly across individual, group, or online settings. Tailoring to cultural context and involving paraprofessionals or community health workers can increase reach, particularly in resource-limited environments. Clinicians are encouraged to address co-occurring conditions–depression, anxiety, sleep disturbance, and grief–through a holistic approach that combines therapy with psychoeducation, stress management, and links to social support. At the same time, providers should maintain self-care to reduce the risk of burnout in high-demand disaster contexts.

Future research should prioritize rigorous controlled trials in post-earthquake settings, including direct comparisons of intervention types and culturally adapted enhancements of established therapies. Longitudinal studies are needed to determine the durability of benefits beyond the short term, while broader outcomes such as functioning, quality of life, and social reintegration require systematic assessment. Innovative delivery formats, such as brief early interventions, mobile-based tools, and family or community-level approaches, also warrant evaluation. Finally, research should expand to include children, adolescents, first responders, and cross-disaster comparisons to improve generalizability and inform evidence-based policy and practice worldwide.

## Conclusion

This systematic review and meta-analysis demonstrates that psychological interventions implemented after the 2023 Kahramanmaraş earthquakes produced significant benefits for adult survivors. Across nine studies, structured interventions–particularly trauma-focused CBT, psychoeducation-based programs, and several culturally informed approaches–were consistently associated with reductions in PTSD, depression, and anxiety symptoms. Group-based delivery and telehealth modalities also appeared feasible and effective during periods when access to traditional services was disrupted. Although the evidence base remains limited, heterogeneous, and concentrated largely on short-term outcomes, the findings reinforce the important role of structured psychosocial interventions in post-disaster mental health care. Importantly, follow-up assessments varied widely across studies (1–12 months), and most data were derived from short-term follow-ups; therefore, statements regarding sustained gains must be interpreted cautiously. Evidence for longer-term durability remains preliminary, and additional trials with standardized follow-up periods are needed. The effects observed for culturally adapted therapies should likewise be interpreted as promising but tentative, given the small number of relevant trials and the absence of moderator or subgroup analyses. Addressing gaps in long-term follow-up, functional outcomes, sleep and substance-use domains, and extending research to children, adolescents, and first responders will be essential for a more comprehensive understanding of post-earthquake mental health needs. Overall, early, scalable, and contextually sensitive psychological interventions remain a critical component of the public health response to major disasters such as the Kahramanmaraş earthquakes.

## Data Availability

The datasets presented in this study can be found in online repositories. The names of the repository/repositories and accession number(s) can be found below: https://doi.org/10.6084/m9.figshare.30016540.
