# Supplementary material for: Effectiveness of psychological interventions for adult survivors of the 2023 Kahramanmaraş earthquakes: a systematic review and meta-analysis
Source: Front Psychol. 2025 Dec 17;16:1696103. doi: 10.3389/fpsyg.2025.1696103 (PMC12754912; doi:10.3389/fpsyg.2025.1696103)
Supplement: Supplementary file 1 [file Data_Sheet_1.PDF]

Effectiveness of Psychological Interventions on Mental Health Outcomes in Survivors of the 2023 Kahramanmaraş Earthquakes: A Systematic Review and Meta-Analysis

metin Çınaroğlu, Eda Yılmazer

Citation

metin Çınaroğlu, Eda Yılmazer. Effectiveness of Psychological Interventions on Mental Health Outcomes in Survivors of the 2023 Kahramanmaraş Earthquakes: A Systematic Review and Meta-Analysis. Not yet published.

PREVIEW

REVIEW TITLE AND BASIC DETAILS

Review title

Effectiveness of Psychological Interventions on Mental Health Outcomes in Survivors of the 2023 Kahramanmaraş Earthquakes: A Systematic Review and Meta-Analysis

Condition or domain being studied

Posttraumatic Stress Disorder; Anxiety; Depression; Sleep disturbances; Substance Use

The review focuses on the psychological impact of the 2023 Kahramanmaraş earthquakes in Türkiye, with a particular emphasis on common mental health conditions observed in disaster-affected populations. These include post-traumatic stress disorder (PTSD), depression, and anxiety, which are among the most prevalent outcomes following large-scale trauma. Additionally, sleep disturbances and substance use problems frequently co-occur with these conditions and may exacerbate overall psychological distress and functional impairment. By synthesizing evidence from studies evaluating psychological interventions among adult earthquake survivors, this review aims to determine the effectiveness of such interventions in reducing PTSD, depressive and anxiety symptoms, as well as improving sleep and addressing maladaptive substance use. The findings will help inform future post-disaster mental health strategies and guide evidence-based clinical practice.

Rationale for the review

The 2023 Kahramanmaraş earthquakes caused widespread devastation and psychological trauma among affected communities in Türkiye. While numerous psychological interventions have been implemented in the aftermath, their effectiveness has not yet been systematically evaluated in this specific post-disaster context. This review aims to synthesize current evidence on the impact of psychological treatments for PTSD, depression, anxiety, sleep disturbances, and substance use among earthquake survivors. By identifying which interventions are most effective and under what conditions, the review will contribute to the development of evidence-based mental health responses in future disaster settings and fill a critical gap in post-earthquake intervention research.

Review objectives

Primary Objective:

To evaluate the effectiveness of psychological interventions in reducing symptoms of post-traumatic stress disorder (PTSD), depression, anxiety, sleep disturbance, and substance use among adult survivors of the 2023 Kahramanmaraş earthquakes.

Secondary Objectives:

To compare the effectiveness of different types of psychological interventions (e.g., CBT, EMDR, group therapy, religiously-integrated approaches).

To examine whether intervention outcomes vary by delivery format (e.g., individual vs. group, online vs. in-person), duration, or timing post-disaster.

To identify potential moderators of treatment effect, including gender, age, and baseline symptom severity.

Review Questions:

What is the overall effectiveness of psychological interventions in reducing PTSD, depression, anxiety, sleep problems, and substance use among adult survivors of the Kahramanmaraş earthquakes?

Are certain types of psychological interventions more effective than others in this context?

Do treatment effects vary based on delivery mode, intervention duration, or demographic factors?

What methodological quality and risk of bias characterize the existing evidence base?

Keywords
Psychological interventions; Earthquake survivors; Post-traumatic stress disorder; Depression; Anxiety; Sleep disturbance

Country
Türkiye

ELIGIBILITY CRITERIA

Population

Included
The review will include studies involving adults aged 18 years and older who were directly exposed to and affected by the 2023 Kahramanmaraş earthquakes in Türkiye.

Inclusion Criteria:

Adults aged 18 years and older

Individuals directly affected by the 2023 Kahramanmaraş earthquakes in Türkiye

General population samples (not restricted to specific professions or subgroups)

Studies evaluating structured psychological interventions (e.g., CBT, EMDR, psychoeducation, group therapy, religious or culturally adapted interventions)

Quantitative studies reporting pre- and post-intervention outcomes on PTSD, depression, anxiety, sleep disturbance, or substance use

Use of validated psychological scales (e.g., PCL-5, BDI, GAD-7, PSQI)

Peer-reviewed articles published between February 2023 and July 2025

Studies published in English or Turkish

Excluded
Exclusion Criteria

Participants younger than 18 years

Studies focusing on Syrian refugees or non-Turkish populations

Studies limited to specific subgroups (e.g., healthcare workers, first responders, pregnant women, students) unless general population data are reported separately

Qualitative-only studies

Studies lacking a control or comparison group for quantitative effect estimates

Interventions not targeting mental health outcomes (e.g., physical rehabilitation only)

Case studies, opinion pieces, editorials, and conference abstracts without full data

Studies with sample sizes fewer than 30 participants per group

Intervention(s) or exposure(s)

Included

Cognitive Behavioral Therapy; Eye Movement Desensitization And Reprocessing Therapy; Trauma Therapy; Religious practices

To be included, interventions must be structured psychological treatments explicitly aimed at reducing post-traumatic stress, depression, anxiety, sleep disturbance, or substance use symptoms among adult survivors of the 2023 Kahramanmaraş earthquakes. Eligible interventions may include cognitive behavioral therapy (CBT), EMDR, trauma-focused therapy, religious or spiritually integrated psychotherapy, psychoeducation, and other manualized therapies. Interventions must be delivered by trained professionals (e.g., psychologists, psychiatrists, counselors, or social workers) and implemented either individually or in groups, in-person or through remote/online formats. Programs with clear therapeutic components—such as goal-setting, exposure, cognitive restructuring, or relaxation training—are eligible. Studies must report measurable pre- and post-intervention outcomes using validated psychological assessment tools.

Interventions that are informal, non-structured, peer-led, pharmacological only, or not focused on mental health outcomes will be excluded.

Excluded
Interventions will be excluded if they are unstructured, non-therapeutic, or lack a defined psychological framework. Studies focusing solely on pharmacological treatments, physical rehabilitation, general social support without a psychotherapeutic component, or informal peer-led activities will be excluded. Interventions not targeting mental health outcomes—such as those addressing only physical health, housing, or economic recovery—will also be excluded. Additionally, studies that do not report validated psychological outcomes or lack pre- and post-intervention assessments will not be eligible for inclusion.

Comparator(s) or control(s)

Included
PICO tags selected: Waiting list control; Active control

Study design
Both randomized and nonrandomized study types will be included.

Included
The review will include quantitative studies that evaluate the effectiveness of psychological interventions using validated outcome measures. Eligible study designs include randomized controlled trials (RCTs), quasi-experimental studies (e.g., non-randomized controlled trials, controlled before-and-after studies), and pre-post intervention studies with a comparison group. Only peer-reviewed, full-text articles published in English or Turkish will be considered.

Context
This review is set within the context of the 2023 Kahramanmaraş earthquakes, one of the most devastating natural disasters in Türkiye's recent history, which resulted in widespread trauma and psychological distress among affected communities. In the aftermath, various psychological interventions were implemented to address the mental health needs of adult survivors. However, the effectiveness of these interventions has not yet been systematically assessed. This review seeks to synthesize the emerging evidence on post-disaster psychological care to inform clinical practice, disaster response planning, and future policy development in similar humanitarian crises. The findings will be particularly relevant for mental health professionals, policymakers, and emergency response planners working in disaster-prone regions.

SIMILAR REVIEWS

Check for similar records already in PROSPERO
PROSPERO identified a number of existing PROSPERO records that were similar to this one (last check made on 12 July 2025). These are shown below along with the reasons given by that the review team for the reviews being different and/or proceeding.

- Systematic Review and Meta-Analysis of the Psychological Impact of the 2023 Kahramanmaraş Earthquakes: Prevalence, Risk Factors, and Outcomes [published 7 February 2025] [CRD42025644127]. The review was judged **not to be similar**
- Traumatic Experiences in Refugees: A Systematic Review and Meta-analysis on the Efficacy of Psychological

Interventions [published 28 December 2023] [CRD42023494553]. The review was judged **not to be similar**
- Interventions for Post-Intensive Care Syndrome-Family: A Systematic Review and Meta-Analysis [published 10 February 2025] [CRD42025644920]. The review was judged **not to be similar**

TIMELINE OF THE REVIEW

Date of first submission to PROSPERO
This record has not been submitted.

Review timeline
Start date: 21 July 2025. End date: 19 September 2025.

Date of registration in PROSPERO
This record has not been published.

AVAILABILITY OF FULL PROTOCOL

Availability of full protocol
A full protocol has been written and uploaded to PROSPERO. The protocol may be accessed through this link https://www.crd.york.ac.uk/PROSPEROFILES/83bc85f675a9ca7d3e8a9f73b7531294.pdf.

SEARCHING AND SCREENING

Search for unpublished studies
Only published studies will be sought.

Main bibliographic databases that will be searched
The main databases to be searched are *Embase.com*, *PsycInfo*, *PubMed*, *SCI - Science Citation Index*, *SSCI - Social Science Citation Index* and *Scopus*.

Search language restrictions
The review will only include studies published in English and Turkish.

Search date restrictions
There are no search date restrictions.

Other methods of identifying studies
Other studies will be identified by: *contacting authors or experts*, *reference list checking* and *searching trial or study registers*.

Link to search strategy
A full search strategy is available in the full protocol as described in the *Availability of full protocol* section

Selection process
Studies will be screened independently by at least two people (or person/machine combination) with a process to resolve differences.

Other relevant information about searching and screening
None

DATA COLLECTION PROCESS

Data extraction from published articles and reports
Data will be extracted independently by at least two people (or person/machine combination) with a process to resolve differences.

Authors will not be contacted for further information.

Study risk of bias or quality assessment

Risk of bias will be assessed using: *Cochrane RoB-2* and *ROBINS-I*

Data will be assessed independently by at least two people (or person/machine combination) with a process to resolve differences.

Additional information will be sought from study investigators if required information is unclear or unavailable in the study publications/reports.

Reporting bias assessment
Risk of bias due to missing results will be assessed

Certainty assessment
The certainty of the evidence for each main outcome (PTSD, depression, anxiety, sleep disturbance, and substance use) will be assessed using the Grading of Recommendations Assessment, Development and Evaluation (GRADE) approach. Domains assessed will include risk of bias, inconsistency, indirectness, imprecision, and publication bias. Two reviewers will independently rate the certainty of evidence as high, moderate, low, or very low, with discrepancies resolved through discussion or third-party adjudication. Summary of Findings (SoF) tables will be generated for key outcomes to present GRADE ratings alongside pooled effect estimates and 95% confidence intervals. These assessments will support transparent interpretation of the overall strength and reliability of the evidence.

OUTCOMES TO BE ANALYSED

Main outcomes
1. Post-Traumatic Stress Symptoms

Definition: Severity of post-traumatic stress disorder (PTSD) symptoms following the Kahramanmaraş earthquakes

Measurement instruments: PCL-5, IES-R, DSM-5-based scales validated in Turkish

Time points: Post-intervention (primary); follow-up if reported

Effect measure: Standardized mean difference (SMD) or mean difference (MD) with 95% confidence intervals (CIs)

2. Depressive Symptoms

Definition: Severity of depressive symptoms among earthquake survivors

Measurement instruments: Beck Depression Inventory (BDI), PHQ-9, CES-D, DASS-21 depression subscale

Time points: Post-intervention (primary); follow-up if reported

Effect measure: SMD or MD with 95% CIs

3. Anxiety Symptoms

Definition: Severity of general anxiety symptoms

Measurement instruments: Beck Anxiety Inventory (BAI), GAD-7, DASS-21 anxiety subscale

Time points: Post-intervention (primary); follow-up if reported

Effect measure: SMD or MD with 95% CIs

4. Sleep Disturbance

Definition: Problems with sleep quality or insomnia symptoms related to post-trauma stress

Measurement instruments: Pittsburgh Sleep Quality Index (PSQI), Insomnia Severity Index (ISI), sleep items within broader symptom checklists

Time points: Post-intervention (primary); follow-up if reported

Effect measure: SMD or MD with 95% CIs

5. Substance Use Symptoms

Definition: Frequency or severity of alcohol or substance misuse following the disaster

Measurement instruments: AUDIT, DAST, ASSIST, or other validated tools

Time points: Post-intervention (primary); follow-up if reported

Effect measure: SMD, MD, or odds ratios (ORs) for dichotomous outcomes, with 95% CIs

Additional outcomes

1. Psychological Well-being or General Mental Health

Definition: Broader emotional or psychological well-being not specific to PTSD, depression, or anxiety

Measurement instruments: GHQ-12, WHO-5 Well-Being Index, MHI-5, or other validated tools

Time points: Post-intervention (primary); follow-up if available

Effect measure: Standardized mean difference (SMD) or mean difference (MD) with 95% confidence intervals (CIs)

2. Resilience or Coping Ability

Definition: Capacity to adapt positively following trauma

Measurement instruments: Connor-Davidson Resilience Scale (CD-RISC), Brief Resilience Scale (BRS), or similar validated measures

Time points: Post-intervention (primary); follow-up if available

Effect measure: SMD or MD with 95% CIs

3. Functional Impairment or Daily Functioning

Definition: Limitations in social, occupational, or general functioning due to psychological symptoms

Measurement instruments: WHODAS 2.0, Sheehan Disability Scale (SDS), or other validated functioning assessments

Time points: Post-intervention (primary); follow-up if available

Effect measure: SMD or MD with 95% CIs

4. Quality of Life

Definition: Overall life satisfaction or perceived quality of health and well-being

Measurement instruments: WHOQOL-BREF, SF-36, EQ-5D

Time points: Post-intervention (primary); follow-up if available

Effect measure: SMD or MD with 95% CIs

PLANNED DATA SYNTHESIS

Strategy for data synthesis

Where sufficient data are available (≥2 studies reporting the same outcome), we will conduct a quantitative synthesis (meta-analysis) using a random-effects model, which is appropriate given the expected clinical and methodological heterogeneity across studies. Separate meta-analyses will be performed for each primary and additional outcome (PTSD, depression, anxiety, sleep disturbance, substance use, and others). Pooled effect sizes will be reported as standardized mean differences (SMD) with 95% confidence intervals (CIs). For dichotomous outcomes, odds ratios (ORs) will be used.

Heterogeneity will be assessed using the I^2 statistic, with thresholds of 25%, 50%, and 75% interpreted as low, moderate, and high heterogeneity, respectively. τ^2 will also be calculated to quantify between-study variance. Where high heterogeneity is detected, potential sources will be explored via subgroup analyses and meta-regression, if sufficient studies are available (≥10 studies per subgroup). Planned subgroup analyses will include intervention type (e.g., CBT, EMDR), delivery format (group vs. individual, online vs. in-person), timing of intervention (early vs. delayed), and participant characteristics (e.g., gender).

Sensitivity analyses will be conducted by excluding studies at high risk of bias to examine the robustness of pooled results. Where meta-analysis is not feasible due to limited or heterogeneous data, findings will be reported narratively, structured around intervention type and outcome domain.

All analyses will be conducted using R (meta, metafor packages) or RevMan 5.4, and visual outputs (forest plots, funnel plots) will be generated accordingly. Funnel plots and Egger's test will be used to examine potential publication bias

when ≥10 studies are available for an outcome.

CURRENT REVIEW STAGE

Stage of the review at this submission

Review stage	Started	Completed
Pilot work	✓	
Formal searching/study identification		
Screening search results against inclusion criteria		
Data extraction or receipt of IPD		
Risk of bias/quality assessment		
Data synthesis		

Review status
The review is currently planned or ongoing.

Publication of review results
Results of the review will be published in English.

REVIEW AFFILIATION, FUNDING AND PEER REVIEW

Review team members
Dr metin Çınaroğlu (review guarantor and contact) ORCID: 0000-0001-6342-3949. İstanbul Nişantaşı University. Türkiye.

No conflict of interest declared.

Dr Eda Yılmazer. ORCID: 0009-0009-3377-5025. Beykoz University. Türkiye.

No conflict of interest declared.

Named contact
Dr metin Çınaroğlu (metincinaroglu@gmail.com). ORCID: 0000-0001-6342-3949. İstanbul Nişantaşı University. Türkiye.

Review affiliation
İstanbul Nişantaşı University

Beykoz University

Funding source
Review has no funding and no agreed support from an academic institution and is done in authors' own time.

Peer review
The protocol was reviewed by members of the study team and academic colleagues with expertise in disaster mental health and systematic review methodology. Feedback was incorporated prior to PROSPERO registration.

ADDITIONAL INFORMATION

Review conflict of interest
Declared individual interests are recorded under team member details.. No additional interests are recorded for this review.

Medical Subject Headings
Adult; Anxiety; Cognitive Restructuring; Counselors; Demography; Depression; Earthquakes; Eye Movement Desensitization Reprocessing; Goals; Humans; Mental Health; Outcome Assessment, Health Care; Psychiatrists; Psychosocial Intervention; Psychotherapy; Psychotherapy, Group; Sleep; Sleep Wake Disorders; Social Workers; Stress

Disorders, Post-Traumatic; Substance-Related Disorders; Survivors

PROSPERO version history

No preview available

Disclaimer

The content of this record displays the information provided by the review team. PROSPERO does not peer review registration records or endorse their content.

PROSPERO accepts and posts the information provided in good faith; responsibility for record content rests with the review team. The guarantor for this record has affirmed that the information provided is truthful and that they understand that deliberate provision of inaccurate information may be construed as scientific misconduct.

PROSPERO does not accept any liability for the content provided in this record or for its use. Readers use the information provided in this record at their own risk.

Any enquiries about the record should be referred to the named review contact
