# Supplementary material for: Effectiveness of psychological interventions for adult survivors of the 2023 Kahramanmaraş earthquakes: a systematic review and meta-analysis
Source: Front Psychol. 2025 Dec 17;16:1696103. doi: 10.3389/fpsyg.2025.1696103 (PMC12754912; doi:10.3389/fpsyg.2025.1696103)
Supplement: Supplementary file 2 [file Supplementary_file_1.docx]

**Supplementary Material 1: Full Search Strings**

**PubMed / MEDLINE**

("Earthquakes"[MeSH] OR earthquake* OR "Kahramanmaraş" OR "Kahramanmaras" OR Türkiye OR Turkey)

AND ("Psychological Intervention" OR "Psychotherapy"[MeSH] OR "Cognitive Behavioral Therapy"[MeSH] OR "CBT" OR "TF-CBT" OR "Trauma-Focused Therapy" OR EMDR OR "Eye Movement Desensitization and Reprocessing" OR Psychoeducation OR "Group Therapy"[MeSH] OR "Religious intervention" OR "Spiritually oriented therapy" OR "Islamic cognitive behavioral therapy")

AND ("Post-Traumatic Stress Disorders"[MeSH] OR PTSD OR "Post-traumatic stress" OR Depression OR "Depressive Disorder"[MeSH] OR Anxiety OR "Anxiety Disorders"[MeSH] OR "Sleep disturbance" OR Insomnia OR "Substance-Related Disorders"[MeSH] OR "Substance use")

Filters: Publication date = Feb 2023 – Jun 2025; Languages = English, Turkish.

**Scopus**

(TITLE-ABS-KEY (earthquake* OR "Kahramanmaraş" OR "Kahramanmaras" OR Türkiye OR Turkey))

AND (TITLE-ABS-KEY ("psychological intervention" OR psychotherapy OR "cognitive behavioral therapy" OR CBT OR "trauma-focused therapy" OR EMDR OR "eye movement desensitization and reprocessing" OR psychoeducation OR "group therapy" OR "religious intervention" OR "spiritually oriented therapy" OR "Islamic cognitive behavioral therapy"))

AND (TITLE-ABS-KEY ("post-traumatic stress disorder" OR PTSD OR "post-traumatic stress" OR depression OR "depressive symptoms" OR anxiety OR "anxiety symptoms" OR "sleep disturbance" OR insomnia OR "substance use"))

AND (LIMIT-TO (PUBYEAR, 2023) OR LIMIT-TO (PUBYEAR, 2024) OR LIMIT-TO (PUBYEAR, 2025))

AND (LIMIT-TO (LANGUAGE, "English") OR LIMIT-TO (LANGUAGE, "Turkish"))

**Web of Science (WoS)**

TS=(earthquake* OR "Kahramanmaraş" OR "Kahramanmaras" OR Türkiye OR Turkey)

AND TS=("psychological intervention" OR psychotherapy OR "cognitive behavioral therapy" OR CBT OR "trauma-focused therapy" OR EMDR OR "eye movement desensitization and reprocessing" OR psychoeducation OR "group therapy" OR "religious intervention" OR "spiritually oriented therapy" OR "Islamic cognitive behavioral therapy")

AND TS=("post-traumatic stress disorder" OR PTSD OR "post-traumatic stress" OR depression OR "depressive symptoms" OR anxiety OR "anxiety symptoms" OR "sleep disturbance" OR insomnia OR "substance use")

Refined by: Document types = Article; Publication years = 2023–2025; Languages = English, Turkish.

**DergiPark** *(Turkish National Database)*

("deprem" OR earthquake* OR "Kahramanmaraş" OR Kahramanmaras OR Türkiye OR Turkey)

AND ("psikolojik müdahale" OR "psikoterapi" OR "bilişsel davranışçı terapi" OR CBT OR "travma odaklı terapi" OR EMDR OR "göz hareketleriyle duyarsızlaştırma ve yeniden işleme" OR "psiko-eğitim" OR "grup terapisi" OR "dini müdahale" OR "manevi terapi" OR "İslami bilişsel davranışçı terapi")

AND ("travma sonrası stres bozukluğu" OR TSSB OR "travma sonrası stres" OR depresyon OR "depresif belirtiler" OR anksiyete OR "kaygı bozuklukları" OR "uyku bozukluğu" OR insomnia OR "madde kullanımı")

Limit: Publication years 2023–2025; Language = Turkish, English.
