# Supplementary material for: Effectiveness of psychological interventions for adult survivors of the 2023 Kahramanmaraş earthquakes: a systematic review and meta-analysis
Source: Front Psychol. 2025 Dec 17;16:1696103. doi: 10.3389/fpsyg.2025.1696103 (PMC12754912; doi:10.3389/fpsyg.2025.1696103)
Supplement: Supplementary file 3 [file Supplementary_file_2.docx]

**Full Characteristics of Included Studies (Extended Data Extraction)**

**Abbreviations:** RCT = randomized controlled trial; QE = quasi-experimental; WL = wait-list; PCL-5/C = PTSD Checklist (DSM-5/civilian); DSTBS = Scale for Determining the Level of Post-Earthquake Trauma; DASS-21 = Depression Anxiety Stress Scales; PHQ-SADS = Patient Health Questionnaire—Somatic, Anxiety, Depressive Symptoms; CERS = Cognitive Emotion Regulation Scale; PTGI = Post-traumatic Growth Inventory; PTCI = Post-traumatic Cognitions Inventory; MRC = Religious Coping (Muslim) Scale; GHQ-12 = General Health Questionnaire. **NR** = not reported.

| **Study (year)** | **Design & sample** | **Recruitment / setting** | **Baseline symptom levels (group means where available)** | **Intervention (format, dose) & Fidelity** | **Comparator** | **Attrition** | **Follow-up** | **Funding / COI** |
| --- | --- | --- | --- | --- | --- | --- | --- | --- |
| **Kafes et al. 2024** | RCT; N=34 (VR n=17; WL n=17). Adults (15–72 y). | Survivors housed in Nazilli municipal dormitory; recruited via municipality/city council briefing; randomized with equal group size; inclusion DSTBS ≥52. | **Pre-test**: DSTBS 67.53 (VR) vs 62.59 (WL); PTGI 61.76 vs 65.59; coping subscales reported (Table 3). | **VR-supported 5-stage protocol** (normalization, reinterpretation, safe place, problem-focused coping, social support); completed within 7 days; **Fidelity:** standardized VR animations and scripted audio; no formal fidelity audit reported. | WL | None (no loss of participants). | Post only (7 days after pre-test); no longer-term follow-up. | **Funding:** none; **COI:** none stated. |
| **Çınaroğlu 2025** | RCT (3 arms); N=45 (ITF-CBT 15→14; TF-CBT 15→13; WL 15→14). Highly religious adults. | Screening of 302 adults across 11 provinces (face-to-face); included if PCL-5 ≥33 and high religiosity (OK-RAS ≥30); computerized randomization. | **Pre** PCL-5: ITF 66.8; TF 69.4; WL 66.5. **Mid** (wk 6) and **post** (wk 12) also reported (Table 2). | **12 weekly 1:1 online sessions** (TF-CBT vs Islamic TF-CBT). **Fidelity:** therapists (≥100 h CBT + 50 h Islamic-CBT training); supervision; external committee rated anonymized audio segments for adherence/competence. | WL | 1–2 per arm (final N above). | Mid-treatment and post-treatment; **no long-term** outcomes in this paper (3–12 m planned). | **Funding:** none; **COI:** none. |
| **Kızılgeçit et al. 2024** | RCT; N=60 adults (3 cities). | Community adults (20–60 y) from Malatya, Elazığ, Kahramanmaraş; randomized to spiritually oriented logotherapy vs WL; site-based delivery. | **Pre**: PTSD (PCL-5) and DSTBS collected; arm-level baseline means NR in abstract; groups described as comparable. | **Logotherapy, 6 weekly group sessions** focusing on meaning-making; **Fidelity:** standardized curriculum; facilitator qualifications NR. | WL | NR (implied minimal). | Immediate post only; no long-term follow-up. | **Funding/COI:** NR. |
| **Çapar & Çuhadar 2025** | RCT; N=61 emerging adults (18–29 y). | Community/university setting (earthquake-affected region); randomized to CBT-based psychoeducation (group) vs WL. | DASS-21 and CERS at baseline; groups similar; arm-level numeric baselines NR in text. | **Group CBT-based psychoeducation** (2×/week; 9 sessions). **Fidelity:** structured manual; trained facilitators; supervision NR. | WL | Minimal/NR. | Immediate post; no longer-term follow-up. | **Funding/COI:** NR. |
| **Toprak et al. 2025** | QE (non-randomized) with untreated comparison; N=24 (exp 11→7; ctrl 11→7 analyzable at 1-y due to missing). | Open call via social media; adults 18–65; allocation by treatment timing preference (Ramadan vs later). | **Experimental group** PCL-5 **pre** 56.36; **post** 51.79; **1-wk** 30.00; **1-mo** 19.86; **1-y** 32.36 (Table 4). PTCI/PTGI/MRC also reported. | **Religiously adapted Brief CBT**, 5 individual sessions over 2.5 weeks; **Fidelity:** therapists trained in model; supervised by model developer. | No-treatment concurrent comparison. | 2 dropped during active phase; five with missing 1-y data. | **Follow-ups at 1 week, 1 month, 1 year.** | **Funding:** none; **COI:** none. |
| **Sezgin & Karagülmez 2025** | Mixed: Study-1 survey (N=239); Study-2 small RCT (N=18; 9 vs 9). | University students; volunteer sign-up; randomization in Study-2. | Baseline PDS, PHQ-SADS, PANAS collected; small-N baseline equivalence implied; numeric means NR. | **BASIC-PH group counseling**, 6 sessions (Study-2); single-session guidance in Study-1. **Fidelity:** protocolized; facilitator qualifications NR. | WL (Study-2). | NR (Study-2 retained N=18). | Post only (semester-length); no long-term follow-up. | **Funding/COI:** NR. |
| **Çakmak et al. 2025** | RCT; N=40 nursing students (20 vs 20). | University cohort (Gümüşhane); volunteers; random assignment; baseline groups similar except residence. | BDI, UCLA Loneliness, Beck Hopelessness; baseline equivalence reported except one demographic; arm-level baseline means NR. | **Trauma psychoeducation**, 6 sessions (coping skills, self-help). **Fidelity:** structured curriculum; fidelity audit NR. | No-intervention control. | NR (appears none). | Post only; no follow-up. | **Funding/COI:** NR. |
| **Gareayaghi et al. 2025** | Cohort (pre-post, no control); N=153 (143 at 6 m). | Telepsychiatry disaster clinic; adults with GHQ-12 ≥13; ≥2 remote sessions (psychoeducation + meds as needed). | **1-mo:** PCL-C 42.47; BDI 36.39. **6-mo:** PCL-C 33.02; depression remained severe on average. | **Telepsychiatry**, individualized care; **Fidelity:** routine clinical supervision; standardized dose NR. | — | 10 lost to follow-up (143/153 completed 6-mo). | **6 months** post-baseline. | **Funding:** APC paid by author; **COI:** none. |
| **İme 2024/25** | RCT (3 arms); N=83 randomized (Online CBT=28; F2F CBT=28; WL=27). | Surveyed 245 earthquake survivors ~1.5 months post-event; eligible adults randomized to three arms. | DASS-21 (Dep/Anx/Stress) & Brief Resilience at baseline (groups equivalent). | **Group CBT counseling**, 8 sessions (online vs face-to-face). **Fidelity:** single trained facilitator; protocolized content; supervision reported. | WL (later crossed-over). | No attrition reported (all completed); per-protocol/ITT NR. | **Immediate post and 2-month follow-up** (WL later treated, “catch-up” demonstrated). | **Funding:** none; **COI:** none. |
