# Supplementary material for: Effectiveness of psychological interventions for adult survivors of the 2023 Kahramanmaraş earthquakes: a systematic review and meta-analysis
Source: Front Psychol. 2025 Dec 17;16:1696103. doi: 10.3389/fpsyg.2025.1696103 (PMC12754912; doi:10.3389/fpsyg.2025.1696103)
Supplement: Supplementary file 4 [file Supplementary_file_3.docx]

Risk of Bias Assessment for Randomized Controlled Trials (RoB-2)

| **Study** | **Randomization Process** | **Deviations from Intended Interventions** | **Missing Outcome Data** | **Measurement of Outcomes** | **Selection of the Reported Result** | **Overall Risk** |
| --- | --- | --- | --- | --- | --- | --- |
| **Kafes et al. (2024)** – VR | Low risk (random allocation reported, balance achieved) | Low risk (protocol standardized, single session) | Low risk (no attrition) | Some concerns (self-report only, not blinded) | Low risk (outcomes prespecified) | **Some concerns** |
| **Çınaroğlu (2025)** – Islamic vs Standard TF-CBT | Low risk (computerized randomization, balanced groups) | Low risk (fidelity checks, supervision reported) | Low risk (minor attrition handled) | Low risk (validated PCL-5, blinded assessor not stated) | Low risk | **Low risk** |
| **Kızılgeçit et al. (2024)** – Logotherapy | Low risk (randomization across sites, equal baselines) | Some concerns (group sessions, therapist not blinded) | Low risk (minimal missing data) | Some concerns (self-reported PTSD, not blinded) | Low risk | **Some concerns** |
| **Çapar & Çuhadar (2025)** – CBT Psychoeducation | Low risk (random assignment, described) | Low risk (structured protocol) | Low risk (minimal attrition) | Some concerns (outcomes self-report, no blinding) | Low risk | **Some concerns** |
| **Sezgin & Karagülmez (2025)** – BASIC-PH Group Counseling | Some concerns (simple randomization, small N=18, baseline imbalance possible) | Low risk | Low risk | High risk (very small sample, outcomes may be unstable) | Some concerns (selective reporting not clear) | **High risk** |
| **Çakmak et al. (2025)** – Trauma Psychoeducation (students) | Some concerns (randomization process not well described) | Low risk (fixed 6-session program) | Low risk | Some concerns (self-reported outcomes, assessor not blinded) | Some concerns (reporting not preregistered) | **Some concerns** |
| **İme (2024/25)** – Online vs Face-to-Face CBT | Low risk (3-arm RCT, clear randomization) | Low risk (protocol fidelity monitored) | Low risk (attrition handled, ITT analysis) | Some concerns (no blinded assessors, self-report) | Low risk | **Low risk** |

**Risk of Bias Assessment for Non-Randomized Studies (ROBINS-I)**

| **Study** | **Confounding** | **Selection of Participants** | **Classification of Interventions** | **Deviations from Intended Interventions** | **Missing Data** | **Measurement of Outcomes** | **Selection of Reported Result** | **Overall Risk** |
| --- | --- | --- | --- | --- | --- | --- | --- | --- |
| **Toprak et al. (2025)** – Religiously adapted Brief CBT | Serious risk (non-randomized, possible confounders like religiosity & baseline PTSD not controlled) | Moderate risk | Low risk (intervention well-defined) | Low risk | Low risk (minor attrition) | Some concerns (self-report only) | Some concerns (not preregistered) | **Serious risk** |
| **Gareayaghi et al. (2025)** – Telepsychiatry Cohort | Serious risk (no control group, high likelihood of natural recovery confound) | Moderate risk (self-selected help-seekers) | Low risk | Low risk | Low risk (follow-up fairly complete) | Some concerns (self-report only, not blinded) | Some concerns | **Serious risk** |
