# Supplementary material for: Effectiveness of psychological interventions for adult survivors of the 2023 Kahramanmaraş earthquakes: a systematic review and meta-analysis
Source: Front Psychol. 2025 Dec 17;16:1696103. doi: 10.3389/fpsyg.2025.1696103 (PMC12754912; doi:10.3389/fpsyg.2025.1696103)
Supplement: Supplementary file 5 [file Supplementary_file_4.docx]

GRADE Evidence Profile / Summary of Findings

**Population:** Adults (≥18 y) directly affected by the 6 Feb 2023 Kahramanmaraş earthquakes
**Interventions:** TF-CBT (standard or Islamic), CBT-based psychoeducation/group CBT, VR-assisted trauma work, spiritually oriented/logotherapy, telepsychiatry
**Comparators:** Wait-list, no treatment, or usual care
**Settings:** Türkiye, 2023–2025

| **Outcome** | **Studies (design) included in certainty rating*** | **Participants (approx.)** | **Pooled effect (post-treatment)** | **Heterogeneity** | **Certainty of evidence (GRADE)** | **Reasons for downgrading / notes** |
| --- | --- | --- | --- | --- | --- | --- |
| **PTSD symptoms** | 4 RCTs (5 comparisons) | ~170 | Hedges’ g = –2.60 (95% CI –4.00 to –1.30) | I² = 86% | **Low** | Downgraded for **risk of bias** (self-report; unclear blinding in several trials) and **inconsistency** (very high heterogeneity). Imprecision not downgraded (CIs exclude 0), but total N small. Non-randomized evidence (Toprak; telepsychiatry cohort) supports direction but not included in rating. |
| **Depression** | 3 RCTs (4 comparisons) | ~170 | Hedges’ g = –1.27 (95% CI –1.73 to –0.81) | I² = 49% | **Moderate** | Downgraded for **risk of bias** (self-report outcomes, assessors not blinded in most). Inconsistency moderate; CIs precise; no serious indirectness. |
| **Anxiety** | 3 RCTs (3 comparisons) | ~170 | Hedges’ g = –1.18 (95% CI –1.55 to –0.82) | I² = 0% | **Moderate** | Downgraded for **risk of bias** (self-report; unclear blinding). No serious inconsistency or imprecision. |

* Only randomized evidence was used for the GRADE ratings shown here. Non-randomized studies (Toprak et al., quasi-experimental; Gareayaghi et al., cohort) are summarized narratively in the manuscript and support the beneficial direction of effect but begin at “low” certainty in GRADE and were not pooled for the certainty ratings.

**Explanatory footnotes (apply across outcomes)**

1. **Risk of bias (RoB-2):** Several RCTs relied exclusively on self-report symptom scales (PCL-5, DASS-21, PHQ-SADS, DSTBS), and outcome assessor blinding was generally not reported; one very small RCT (n=18) had high risk.
2. **Inconsistency:** PTSD showed **very high heterogeneity** (I² = 86%) with diverse interventions (VR, logotherapy, TF-CBT variants) and formats; depression showed **moderate** heterogeneity (I² = 49%); anxiety had **low** heterogeneity (I² = 0%).
3. **Indirectness:** Direct population (adult survivors of the 2023 earthquakes), direct interventions and outcomes; not downgraded.
4. **Imprecision:** CIs exclude the null across outcomes; total N is modest but effects are large; not downgraded.
5. **Publication bias:** Not assessable with <10 studies per outcome; no clear signals, but undetected bias cannot be excluded (no downgrade).
6. **Upgrading:** Not applied (RCT bodies of evidence start at “high”; downgrades applied as above).

**One-sentence SoF summary (for the main text, if useful)**

Compared with wait-list/usual care, structured psychological interventions delivered to adult survivors of the Kahramanmaraş earthquakes produced **large reductions** in **PTSD, depression, and anxiety** at post-treatment; the **certainty of evidence** is **low** for PTSD (due to heterogeneity and RoB) and **moderate** for depression and anxiety.
